# Licensing and niche competition in spermatogenesis: mathematical models suggest complementary regulation of tissue maintenance

**DOI:** 10.1242/dev.202796

**Published:** 2025-01-02

**Authors:** Rodrigo García-Tejera, Jing-Yi Tian, Marc Amoyel, Ramon Grima, Linus J. Schumacher

**Affiliations:** ^1^ Centre for Regenerative Medicine, Institute for Regeneration and Repair, University of Edinburgh, Edinburgh EH16 4UU, UK; ^2^ Department of Cell and Developmental Biology, University College London, London WC1E 6BT, UK; ^3^ School of Biological Sciences, University of Edinburgh, Edinburgh EH9 3BF, UK

**Keywords:** Cell heterogeneity, Competition for niche access, Spermatogenesis, Stem cell licensing, Stem cells, Stochastic modelling

## Abstract

To maintain and regenerate adult tissues after injury, division and differentiation of tissue-resident stem cells must be precisely regulated. It remains elusive which regulatory strategies prevent exhaustion or overgrowth of the stem cell pool, whether there is coordination between multiple mechanisms, and how to detect them from snapshots. In *Drosophila* testes, somatic stem cells transition to a state that licenses them to differentiate, but remain capable of returning to the niche and resuming cell division. Here, we build stochastic mathematical models for the somatic stem cell population to investigate how licensing contributes to homeostasis. We find that licensing, in combination with differentiation occurring in pairs, is sufficient to maintain homeostasis and prevent stem cell extinction from stochastic fluctuations. Experimental data have shown that stem cells are competing for niche access, and our mathematical models demonstrate that this contributes to the reduction in the variability of stem cell numbers but does not prevent extinction. Hence, a combination of both regulation strategies, licensing with pairwise differentiation and competition for niche access, may be needed to reduce variability and prevent extinction simultaneously.

## Teaser

**Summary:** Stochastic modelling reveals that combining licensing with pairwise differentiation and niche competition is crucial for maintaining stem cell numbers and for preventing extinction in *Drosophila* testes.

## INTRODUCTION

In most tissues, formation, maintenance and regeneration rely on a hierarchical structure in which stem cells, at the apex of the lineage tree, produce differentiating offspring (Laurenti and Göttgens, 2018; Moore and Lemischka, 2006). To do so, a multitude of plausible strategies to regulate their population size have been proposed, such as competition for niche access (García-Tejera et al., 2022; Corominas-Murtra et al., 2020; Parigini and Greulich, 2024; Kitadate et al., 2019), competition for niche signals (Yoshida, 2018; Kitadate et al., 2019; Jörg et al., 2021; Stine and Matunis, 2013), mechanical feedback (Hannezo et al., 2016; Vining and Mooney, 2017; Zhang et al., 2021) or feedback from the more differentiated populations (Renardy et al., 2018; Lander et al., 2009; Rodriguez-Brenes et al., 2013). However, it is still unclear how to distinguish the presence or absence of different regulation strategies from snapshots of the stem cell populations or clonal sub-populations, which are what can often be measured experimentally. Recent years have seen an increasing number of studies reporting a diverse set of internal states in stem cell populations of different tissues (Amoyel et al., 2016; Yuen et al., 2021; Weinberger et al., 2016; Haug et al., 2008; Nakagawa et al., 2021; e Melo and de Sauvage, 2019; Goodell et al., 2015; Nichols and Smith, 2009; Ying et al., 2008; Takahashi and Yamanaka, 2016; Weinberger et al., 2016). The role of cell state heterogeneity in the regulation of stem cell populations and tissue function remains poorly understood.

**Fig. 1.**
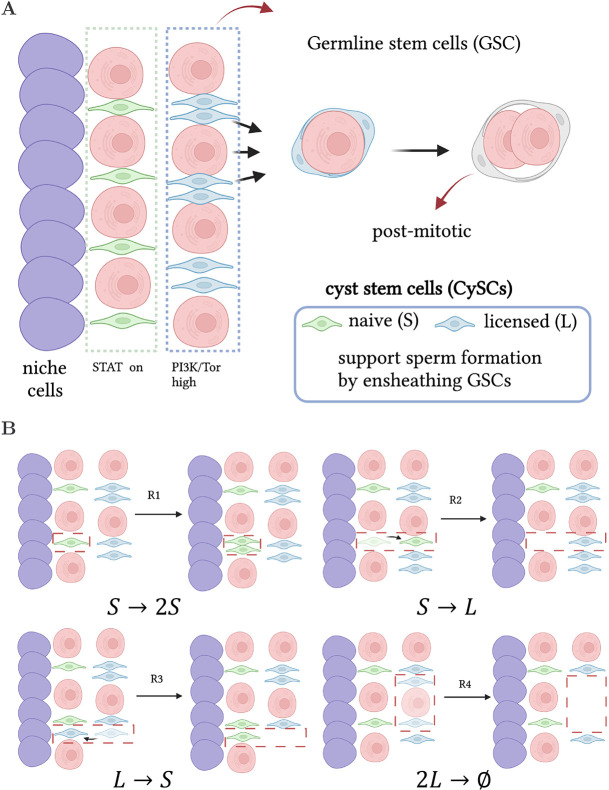
**Modelling CySCs in the *Drosophila* testis.** (A) Schematic of the GSC and CySC niche. GSCs (pink) and CySCs (green) touching the hub (blue cells) have access to pro-self-renewing signals and maintain stemness. CySCs not in contact with the hub lose access to pro-self-renewing signals and become licensed for differentiation, although they require active signalling to differentiate. To form a cyst, two CySC daughters surround one GSC daughter (or gonialblast). These co-differentiate, whereby the CySC daughters become post-mitotic cyst cells, while the germ cell progresses through spermatogonial divisions. (B) Schematic of the four reactions considered in the *SL* model.

A startling example where different internal stem cell states are present is spermatogenesis in *Drosophila*. Spermatogenesis is supported by germline (GSCs) and cyst (CySCs) stem cells (Amoyel et al., 2016; Yuen et al., 2021; Greenspan et al., 2015). Both CySCs and GSCs co-exist in the same niche; a hub consisting of approximately 10 to 12 niche cells providing pro-cell-division signals (Greenspan et al., 2015) (Fig. 1A). GSCs divide into, in most cases, one daughter GSC that remains in contact with the hub and a differentiating progeny. The latter, known as a gonialblast, undergoes four rounds of incomplete division to form a cyst, which later matures into spermatocytes. CySCs, on the other hand, produce post-mitotic differentiating cells, known as cyst cells. Cyst formation begins with a pair of CySCs enveloping a gonialblast, allowing it to progress through differentiation. In turn, the two CySCs differentiate in post-mitotic cyst cells. The regulation mechanisms that promote cyst formation without exhaustion or over-accumulation of GSCs or CySCs remain elusive.

Evidence supports that cyst formation is not regulated by the coordination of GSC and CySC division, but rather at the level of co-differentiation of gonialblasts and CySC daughters (Lenhart and DiNardo, 2015). CySCs in contact with the hub maintain lineage-maintenance ability by receiving signals from the hub through the Janus Kinase and Signal Transducer and Activator of Transcription (JAK/STAT) pathway, leading to expression of the transcription factor zinc-finger homeodomain 1 (Zfh1), which is necessary and sufficient for self-renewal (Leatherman and DiNardo, 2008). During differentiation of CySC daughters, Zfh1 expression is downregulated and the cyst cell marker Eyes Absent (Eya) is upregulated (Fabrizio et al., 2003). Such commutation of expression levels is triggered by signalling through the Phosphoinositide 3-kinase (PI3K)/Target of Rapamycin (Tor) pathway, which is high for CySCs that are not in contact with the hub (Amoyel et al., 2016). Indeed, knocking down PI3K or Tor pathways results in cells remaining Zfh1^+^ Eya^−^, confirming that differentiation requires active signalling (Amoyel et al., 2014; Yuen et al., 2021). Put together, these findings suggest the existence of at least two subpopulations of CySCs: (1) CySCs in contact with the hub, known as naive CySCs, characterised by high expression of Zfh1 triggered by signalling through the JAK/STAT pathway; and (2) CySCs further away from the hub, known as licensed CySCs, with low JAK/STAT activity but high expression of Zfh1. Stem cells in this latter state are ready for differentiation upon receiving an additional, unknown signal through the PI3K/Tor pathway, but can also fully regain stem cell function should they regain contact with the hub (Amoyel et al., 2016; Yuen et al., 2021).

While the presence of licensed states is thought to play a role in tissue maintenance and regeneration (Amoyel et al., 2014; Nakagawa et al., 2021; Hara et al., 2014), it is not clear to what extent the combined effects of reversible transitions between naive and licensed states, and the irreversible differentiation occurring in cell pairs upon cyst formation serve as a viable regulation strategy for stem cell numbers to recover after injury, or whether additional regulatory mechanisms are necessary. To investigate this, we build a minimal mathematical model (*SL* model) in which stem cells can stochastically proliferate, switch back and forth from naive to licensed stem cell states, and form cysts. Analysis of the *SL* model reveals that licensing combined with pairwise differentiation is a viable strategy to achieve a stable homeostatic state in stem cell populations. We compare our model results with experimental observations of the number of licensed and naive stem cells in adult *Drosophila* testes under homeostatic conditions. Our analysis shows that the size of the fluctuations for the experimental measurements is lower than those predicted by our model, pointing to the likely presence of additional regulation strategies. We build an additional model (v*SL* model) that includes both regulation through licensing and autonomous regulation of the naive population in the form of competition for niche access, because it has been shown to take place in the CySC/GSC niche (Amoyel et al., 2014; Issigonis et al., 2009; Singh et al., 2010). The size of fluctuations in the v*SL* model can match the fluctuations size observed experimentally. We show that, while regulation through licensing and pairwise differentiation alone fails to reduce the fluctuations in the size of the stem cell population, it is extremely effective at preventing the extinction of the stem cell pool. Conversely, regulation through competition for niche access alone succeeds at reducing the fluctuations, but cannot prevent extinction. Our results suggest that, for a tissue to exhibit low stochastic fluctuations in stem cell numbers while simultaneously preventing stochastic extinction, a combination of regulation through licensing with pairwise differentiation and competition for niche access is likely needed.

## RESULTS

### Licensing combined with pairwise differentiation of stem cells is a viable regulation strategy

To analyse the viability of the combination of licensing and pairwise differentiation as a regulation strategy, we built a computational model for stem cell populations that includes both naive and licensed states. The dynamics of stem cell populations has typically been modelled mathematically as critical birth-death processes (Till et al., 1964; García-Tejera et al., 2022; Klein and Simons, 2011; Robertson et al., 2022), which assumes a minimal set of hypotheses: stem cells, *S*, undergo division and differentiation/death stochastically, comprising a set of two stochastic reactions that take place at an equal rate: division *S*→2*S* and differentiation/death 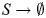. One of the reasons for the success of the critical birth-death process for modelling stem cell populations originates from its ability to reproduce core observations in clonal dynamics, such as the scaling laws relating the number and sizes of surviving clones over time (Klein and Simons, 2011; Robertson et al., 2022; Parigini and Greulich, 2020; Rulands et al., 2018; Corominas-Murtra et al., 2020). The critical birth-death process assumes that a homeostatic state is present (the cell division and differentiation/death rates are equal), but remains agnostic to the mechanism that makes such steady state possible. Recent studies have proposed modifications to the critical birth-death process that include different mechanisms to ensure homeostasis and recovery dynamics (García-Tejera et al., 2022; Kitadate et al., 2019; Greulich and Simons, 2016).

Our model for the population of CySCs can be seen as an extension to the critical birth-death process, with the following minimal set of hypotheses:

(1) There are two types of CySCs: those in contact with the hub, which are proliferative, known as naive stem cells; and those that lose contact with the hub, have no access to niche signals and are ready to differentiate, known as licensed stem cells.

(2) All naive stem cells have the same proliferative potential, can divide stochastically (*R*1 in Fig. 1B) and can also move away from the niche stochastically, losing contact with the hub, thus becoming licensed stem cells (*R*2 in Fig. 1B).

(3) Licensed stem cells, on the other hand, can stochastically move back to the niche and regain contact to the hub, becoming naive stem cells again (*R*3 in Fig. 1B), or they can act in pairs, forming a cyst and thus irreversibly differentiating to a post-mitotic state (*R*4 in Fig. 1B). The frequent migration of licensed stem cells back to the niche is supported by previous work (Chiang et al., 2017) and our lineage-tracing experiment (see supplementary Materials and Methods section ‘Further evidence that de-licensing is a frequent event’ and Fig. S6).

(4) We assume that cell death is much more infrequent than any of the four reactions *R*1−*R*4, and can be disregarded (as supported by Hasan et al., 2015). Taken together, these hypotheses can be captured by the *SL* model, which consists of four stochastic reactions acting upon the naive (*S* species) and licensed (*L* species) populations (Fig. 1B): *S*→2*S* (division), 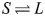 (licensing and de-licensing) and 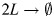 (cyst formation). In the *SL* model, cyst formation only depends on the number of licensed stem cells; hence, we are assuming an abundance of germline stem cells. Licensed stem cells exhibit high motility (Movie 1), which facilitates cyst formation in a well-mixed system. Consequently, the spatial distribution of germline stem cells may not significantly impact cyst formation, supporting our assumption.

A deterministic analysis of the *SL* model, i.e. ignoring the variability of the stem cell numbers due to randomness of the processes involved, reveals that there is a homeostatic state provided that the rate at which naive stem cells proliferate is lower that the rate at which they become licensed (to avoid accumulation of naive stem cells). The parameters of the deterministic *SL* model can be expressed in terms of the average number of naive and licensed stem cells in homeostasis, 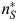 and 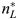, respectively, and the ratio between the average licensing and division rates per cell, *α* (see supplementary Materials and Methods). We can determine the first two parameters from experimental data, obtaining 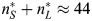 and 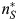 between 13 and 19 (see Materials and Methods). For *α*, however, there is no experimental estimate, other than the observation that it must be larger than 1 to avoid accumulation of naive stem cells. We therefore proceed by studying the behaviour of the *SL* model for plausible values of *α*.

**Fig. 2.**
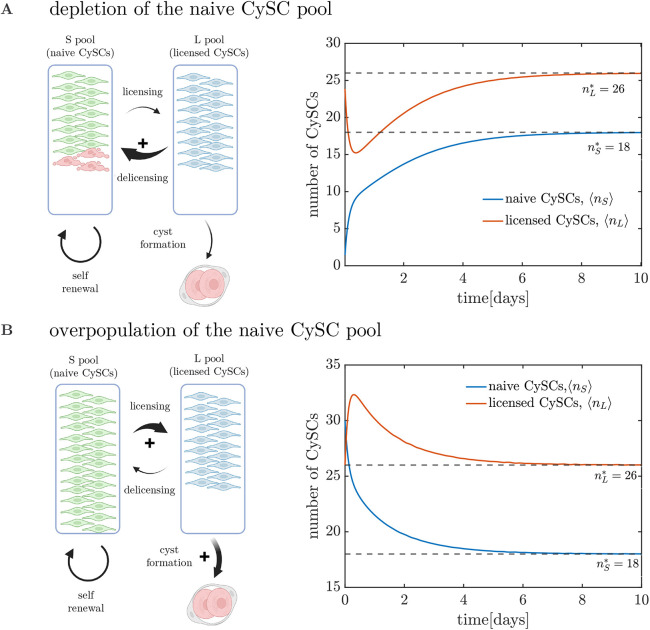
**Response of the deterministic *SL* model to perturbations around cell numbers at homeostasis, given by the steady state** 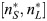
**.** (A) Upon depletion of the naive CySC pool, a net flux of cells from *L* to *S* state takes place, followed by division that restores the homeostatic CySC numbers. (B) When the naive CySC pool is overpopulated, a net flux from the *S* to the *L* pool takes place, along with a larger depletion of the *L* pool due to cyst formation. Parameters are set to 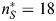 and 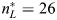 (in accordance with the experimentally observed values) and *α*=3, with initial conditions [*n*_*S*0_, *n*_*L*0_]=[0, 26] for naive CySC depletion (A) and [*n*_*S*0_, *n*_*L*0_]=[30, 26] for naive CySC overpopulation (B).

The *SL* model predicts recovery to the homeostatic state after a perturbation, regardless of the parameter values (see supplementary Materials and Methods). To analyse qualitatively the mechanisms behind the recovery capabilities, we performed *in silico* perturbation experiments. In homeostasis, the number of naive CySCs licensing per unit time is balanced with the number of naive CySCs proliferating plus the number of licensed CySCs de-licensing per unit time. If the number of naive CySCs falls below the homeostatic value, the frequency (total number of events per unit time) of both the division and licensing events decreases, while the frequency of de-licensing remains constant (Fig. 2A, left). As a consequence, a de-licensing flux of cells restores the naive CySC pool numbers. In turn, the licensed CySC pool becomes partially depleted before converging back to its homeostatic value (Fig. 2A, right). Conversely, a surplus of the number of naive CyCSs increases the net flux of naive CySCs licensing, followed by a higher increase in the cyst formation rate that restores the numbers of both the naive and licensed CySCs to homeostatic values (Fig. 2B).

The mechanisms of recovery dynamics presented above require the capability of licensed CySCs to regain naive CySC status. From a mathematical standpoint, the fact that differentiation of licensed stem cells occurs in pairs is the key element that provides the system with stability. However, without de-licensing, such stability cannot be extended to the naive stem cell pool. In an alternative scenario in which CySCs irreversibly license or differentiate after losing contact with the niche, the homeostatic state would be neutrally stable, i.e. the stem cell numbers would not recover after perturbations unless additional regulation strategies were triggered (see supplementary Materials and Methods).

### Licensing with pairwise differentiation prevents stochastic extinction and aids recovery dynamics

**Fig. 3.**
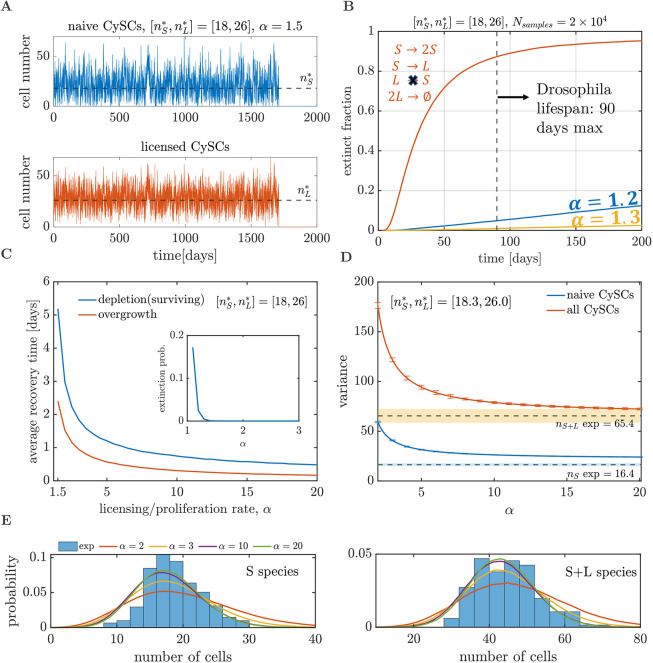
**Stochastic implementation of the *SL* model.** (A) Stochastic trajectories for the naive (blue) and licensed (orange) CySCs simulated via the Gillespie algorithm. Cell numbers fluctuate around their homeostatic values (black dashed lines) until they undergo stochastic extinction. (B) Fraction of extinct tissues as a function of time, over an ensemble of 2×10^4^ simulated tissues, for the *SL* model (blue and yellow lines) and the modified model scenario (orange line), in which naive stem cells irreversibly differentiate upon losing contact with the niche (see section ‘Licensing with pairwise differentiation prevents stochastic extinction and aids recovery dynamics’). (C) Average recovery time (over 2×10^4^ realisations) after depletion (conditional to survival, blue line) or duplication (orange line) of the naive CySC pool, as a function of *α*. The inset shows the probability of extinction after depletion of the naive CySC pool, as a function of *α*. (D,E) Comparison between the outcomes of the *SL* model and experimental observations. (D) Variance of the population sizes (solid lines) predicted by the *SL* model, calculated using Eqn S17 in the supplementary Materials and Methods. Error bars indicate standard error. Dashed lines show average experimental measurements; shaded regions indicate one standard deviation. (E) Experimental (histograms) and *SL* model (solid lines) distributions of naive (left) and total (right) CySC numbers.

The stochastic nature of division, licensing and cyst formation events renders chance extinction of the CySC population plausible. A stochastic implementation of the *SL* model (Gillespie, 1976; Schnoerr et al., 2015) reveals the naive and licensed CySC numbers initially fluctuating around the values of 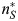 and 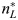, respectively, following a quasi-steady state distribution, but eventually reaching stochastic extinction (Fig. 3A). Note that extinction is certain to take place for all parameter values and it is not predicted when approaching the *SL* model deterministically, as it is a purely stochastic trait. A more-thorough analysis of the onset of extinction dynamics triggered by fluctuations can be found in García-Tejera et al. (2022) and Assaf and Meerson (2010).

After establishing that extinction is certain to take place, the question remains whether the expected extinction time falls within the lifespan of *Drosophila* [median between ∼40 and ∼90 days (Ziehm et al., 2013; Ziehm and Thornton, 2013), although the lifespan is sensitive to temperature and resource availability]. For the purpose of this analysis, we consider a maximum lifespan of 90 days, although our conclusions remain valid for different values. To investigate the extinction times predicted by our model, we simulated, for different values of *α*, an ensemble of 2×10^4^ tissues. Analysis of the cumulative distribution of extinction times, i.e. the fraction of extinct tissues at different times, reveals that fewer than 5% of the trajectories become extinct within the lifespan of *Drosophila*, regardless of the value of *α* (blue and yellow lines in Fig. 3B). For higher values of *α*, the fraction of extinct trajectories within the lifespan of *Drosophila* decreases. Indeed, when *α*>1.3 fewer than 0.1% of trajectories become extinct before 90 days. These results remain valid for all the plausible values of 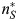 (between 13 and 19).

The importance of reversibly licensed CySCs in preventing stochastic extinction becomes evident when considering an alternative scenario where naive stem cells irreversibly differentiate upon loss of contact with the niche. Consequently, cyst formation would involve a pair of already differentiated cells. Such a scenario can be captured by the reactions *S*→2*S*, *S*→*L* and 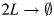 (see supplementary Materials and Methods). Simulation of 2×10^4^ tissues with this model leads to 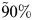 of the stochastic trajectories becoming extinct within the lifespan of *Drosophila* (orange line in Fig. 3B). The striking difference between the fraction of extinct trajectories in the *SL* model with reversible licensing and lack thereof points to a crucial role for licensing in preventing stochastic extinction.

When a perturbation takes place, the recovery time of the CySC population to its homeostatic state is heavily affected by the licensing and de-licensing rates. To show this, we simulated two types of perturbation experiments, depletion and duplication of the naive CySC pool size. The average recovery time across 2×10^4^ realisations in both perturbation experiments decreases steadily as *α* increases (Fig. 3C). Note that for the depletion of the naive CySC pool experiments, we consider the recovery time conditional to survival, since there is a chance that extinction occurs before recovery (inset in Fig. 3C). However, this probability vanishes at very low values (*α*≈1.3). When *α*→1, the average recovery times increase steadily. Indeed, for the case of depletion of the naive CySC pool, the recovery time diverges. To understand this, note that when the naive CySC pool is empty, the system relies on de-licensing events to repopulate it. Since the ratio between the de-licensing to division rate is 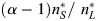 (see supplementary Materials and Methods), letting *α*→1 while keeping 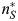 and 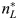 constant leads to the de-licensing rate tending towards 0, making the expected time for de-licensing events tend to infinity. The above analysis of the *SL* model illustrates the benefit of having high licensing/de-licensing rates to minimise the probability of extinction after a perturbation (*α*>1.3 ensures an extinction probability lower than 0.5%) and reduce recovery times.

### Licensing with pairwise differentiation alone cannot account for the fluctuations in the stem cell numbers

To explore the effect of licensing and de-licensing in the fluctuations of the stem cell numbers under homeostatic conditions, we compared the fluctuation size from the experimental counts of the number of licensed and naive CySCs (see Materials and Methods section ‘Measurement of CySC numbers’) with those from the *SL* model for different values of the ratio between the licensing and division rates of the naive CySCs, *α*. In the *SL* model, when the ratio between the licensing and division rates, *α*, increases while maintaining 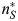 and 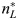 as fixed, the variances of the naive 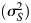 and total 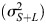 CySC pools decrease steadily (Fig. 3D,E) approaching the asymptotic values 
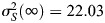
 (standard error, SE=0.3) and 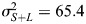 (SE=1.0). For an analytical derivation of the moments of the *SL* model, see supplementary Materials and Methods. These values are larger than the experimentally measured variances 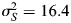 (SE=1.5) and 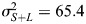 (SE=6.9). In particular, the asymptotic variance of the naive CySC population is 34% higher than its experimental value.

Hence, if the behaviour of the naive and licensed CySC populations is determined solely by stochastic division, licensing/de-licensing and cyst formation, the observed fluctuations should be larger than the ones observed experimentally. We hypothesise that there are additional regulation strategies or restrictions acting on the CySC populations that the *SL* model neglects, such as those that have been shown to take place in different tissues (García-Tejera et al., 2022; Corominas-Murtra et al., 2020; Parigini and Greulich, 2024; Kitadate et al., 2019; Yoshida, 2018).

### Introduction of competition for niche access yields fluctuations matching the experimental values

In the case of the *Drosophila* testes, stem cells are also competing for niche access (Amoyel et al., 2014; Issigonis et al., 2009; Singh et al., 2010). In a model that includes competition for niche access, the propensity for stem cell division depends not only on the number of stem cells present, but also on the space available in the niche; a full niche will offer no opportunity for cell growth before division, thus rendering division less likely, while an empty niche presents no restriction for cell growth and division.

**Fig. 4.**
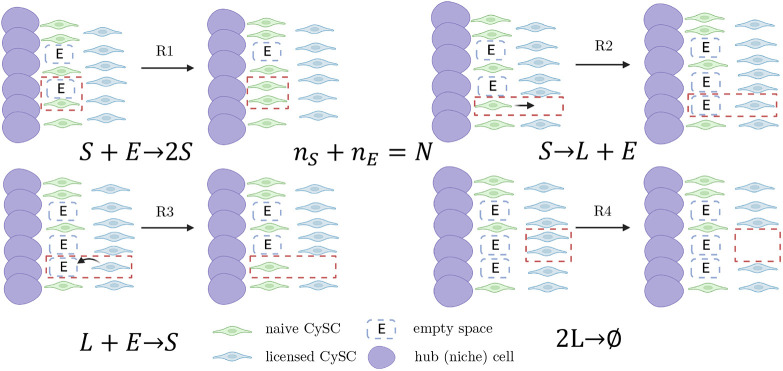
**Constitutive actions of the v*SL* model and their corresponding elements in the reaction network of the model.** R1 is division, R2 is licensing, R3 is de-licensing and R4 is cyst formation.

We built upon the *SL* model to include competition for niche access. An efficient way to model stem cell populations with competition for niche access is the birth-death process with volume exclusion (v*BD*) (García-Tejera et al., 2022). The v*BD* includes a division rate that is dependent on the space available by introducing an empty space species *E* and considering that, for division to take place, a stem cell needs to react with an empty space particle, *S*+*E*→2*S*, an idea introduced by McKane and Newman (2004). Hence, when empty space is abundant, division takes place with a rate proportional to the number of stem cells (assuming mass-action kinetics), and when space is scarce the division rate drops, as also explored by Cianci et al. (2017). We can follow similar ideas to introduce competition for niche access in the *SL* model, resulting in the *SL* model with volume exclusion (v*SL*), which consists of three species: naive CySCs *S*, licensed CySCs *L* and empty space in the niche *E* (Fig. 4). A naive CySC consumes an empty space in the niche to proliferate, *S*+*E*→2*S*. To license, a naive CySC moves away from the niche, losing contact with niche signals, and leaving an empty space, *S*→*L*+*E*. Conversely, when a licensed CySC goes back to the niche and de-licenses, it consumes an empty space, *L*+*E*→*S*. Similarly to the *SL* model, cyst formation is captured by 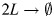. Finally, to account for limited space in the niche, we consider that the number of naive CySCs plus empty spaces is conserved, *n*_*S*_+*n*_*E*_=*N*, where *N* is the carrying capacity (maximum number of cells) of the niche.

**Fig. 5.**
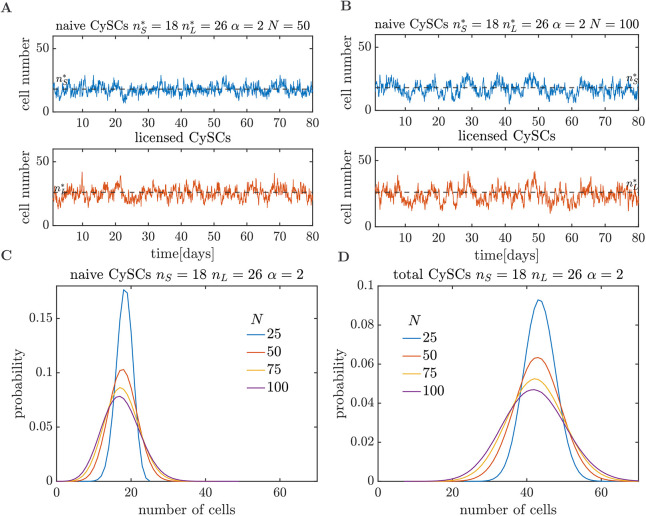
**Quasi-steady state dynamics for the v*SL* model.** (A,B) Stochastic trajectories for the v*SL* model with carrying capacities *N*=50 (A) and *N*=100 (B). (C,D) Quasi-steady state distributions for the naive (C) and total (D) number of CySCs for different carrying capacities.

**Fig. 6.**
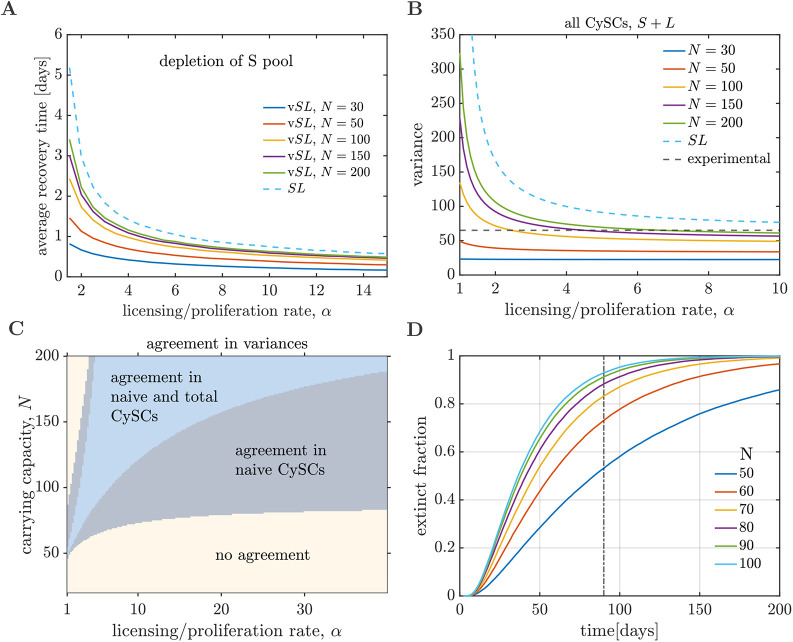
***SL* model with volume exclusion (v*SL*).** (A) Recovery time as a function of the ratio between the licensing and division rates in an empty niche, *α*, for the v*SL* model with different carrying capacities (continuous lines). The recovery times are lower than those observed for the *SL* model (dashed blue line). (B) Homeostatic variance of all (*S*+*L*) CySCs as a function of *α* for different carrying capacities. The variances are always lower than those observed for the *SL* model (blue dashed lines). Experimentally measured variances (black dashed lines) fall within the range of variances of the v*SL* model. Similar observations can be made for the variance of the naive CySCs. (C) Parameter regions of agreement between the experimentally measured variances and the v*SL* model. For parameters in the blue region, both the variance of the naive and total CySCs produced by the v*SL* model fall within one standard error of the corresponding variances measured experimentally. The minimum carrying capacity for the parameters that agree with experimental data is larger than *N*=50. (D) Extinction experiments for the v*SL* model without the de-licensing reaction but maintaining competition for niche access. The extinct fraction is calculated as the cumulative distribution of extinction times over ensembles of 5×10^4^ independent simulations.

Similarly to the *SL* model, the deterministic behaviour of the v*SL* model exhibits a homeostatic state that is characterised by an equilibrium number of naive 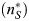 and licensed 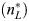 CySCs (Fig. 5). The additional parameters are the ratio between licensing to division rates per cell on an empty niche, *α*, and the carrying capacity *N*. In the limit of the carrying capacity tending towards infinity, *N*→∞, the behaviours of the v*SL* and *SL* models become identical. The homeostatic state of the v*SL* model exhibits recovery dynamics after a perturbation (see supplementary Materials and Methods). The recovery dynamics of the v*SL* model involve the cooperation of two mechanisms: licensing and competition for niche access. As a consequence, the recovery time of the v*SL* model is shorter than in the *SL* model (Fig. 6A).

Combining licensing with pairwise differentiation and competition for niche access as regulation strategies leads to smaller fluctuations in CySC numbers than solely relying on licensing and pairwise differentiation, as shown by stochastic simulations of the v*SL* model (Fig. 6B). Analogous to the *SL* model, the fluctuation size is lower for higher values of *α*. Space availability also affects the size of fluctuations, rendering these smaller for lower carrying capacities *N* (Fig. 6B). Conversely, in the limit of *N*→∞, the distribution of CySC numbers tends towards that of the *SL* model.

We found a region of the parameter space, defined by the carrying capacity *N* and licensing/division rate *α*, where the variances for the naive and total CySC population yielded by the v*SL* model agree with the experimental observations within one standard error (Fig. 6E). In the same region, the variance of the naive CySCs also agrees with experimental values. Although our limitations in the sample sizes prevent us from precisely inferring optimal values of *N* and *α*, our analysis establishes a lower bound for the carrying capacity, which must be larger than 50. A lower carrying capacity leads to fluctuations in the total CySC number, which are lower than the experimentally observed values. Note that the carrying capacity serves as an effective parameter in our model, summarising how space availability influences the rate of division per cell. It should not be interpreted as the actual maximum number of cells that can occupy the stem cell niche.

### Competition for niche access alone leads to fast extinction

Competition for niche access reduces fluctuations in cell number in a scenario where CySCs can reversibly transition between naive and licensed states. The question remains whether competition for niche access alone is capable of sustaining homeostasis and preventing stochastic extinction. To investigate this, we considered an alternative model that includes competition for niche access but assumes that stem cells irreversibly differentiate upon detachment from the niche. Analogous to the section ‘Licensing with pairwise differentiation prevents stochastic extinction and aids recovery dynamics’, we implemented such a scenario by removing the de-licensing reaction in the v*SL* model.

For carrying capacities within the region of agreement between the v*SL* model and the experimental data (i.e. *N*>50, see Fig. 6C), competition for niche access alone leads to fast extinction (Fig. 6D). After simulating an ensemble of 5×10^4^ tissues, the fraction of extinct tissues over time depends on the carrying capacity (Fig. 6D). However, for all plausible carrying capacities, more than 50% of the tissues become extinct within the lifespan of *Drosophila*. In contrast, when de-licensing is present but competition for niche access is absent (*SL* model), fewer than 5% of the tissues become extinct for all parameter regimes (see Fig. 3B). Competition for niche access alone is therefor not capable of preventing stochastic extinction.

### The v*SL* model accurately predicts stem cell recovery dynamics after depletion

We evaluated the predictive capability of the v*SL* model for recovery dynamics by comparing its predictions with the outcomes of an experiment where CySC numbers were depleted. Specifically, we starved flies to reduce the total number of CySCs and re-fed them until they recovered their homeostatic CySC numbers (McLeod et al., 2010) (see Materials and Methods). We counted the number of Zfh1^+^–Eya^−^ CySCs every day during the recovery to determine the dynamics of CySC population recovery from depletion, similarly to Amoyel et al. (2014). In fed controls of the same age, the total number of CySCs was 32.4, with a standard error of 0.9, while in flies starved for 14 days, the number dropped to 21.4, with a standard error of 0.9. We considered that 30% of CySCs are naive in homeostasis, as reported by Amoyel et al. (2014). One limitation of our experiment is that the identity of the remaining CySCs after starvation is unknown. Hence, we considered different scenarios in our model: (1) all the surviving cells were in the licensed state; (2) surviving cells were in both states, but with a higher fraction of cells in the licensed state; (3) the same fraction of each population survived; and (4) most surviving cells were in the naive state. Note that a scenario where all surviving cells are in the naive state is very unlikely, as it exceeds the number of naive CySCs present in homeostatic conditions.

**Fig. 7.**
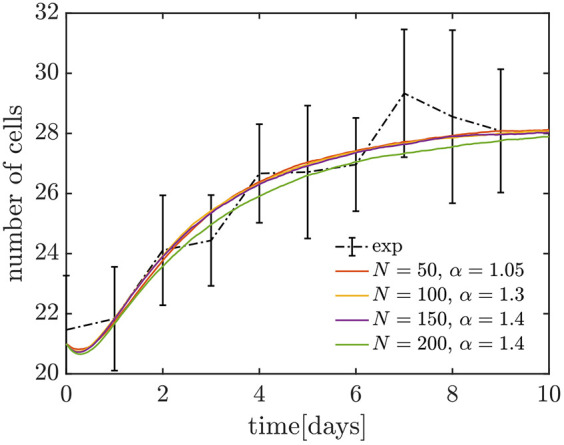
**Recovery dynamics of the CySC population.** Comparison between the starvation and re-feeding experiment (see Materials and Methods; black dot-dash line, error bars represent 95% confidence intervals), and recovery dynamics of the v*SL* model (coloured lines), for different carrying capacities. For each carrying capacity, we chose the values of the ratio between the licensing and division rates, *α*, that provide the best fit to experimental data. In these numerical experiments, we consider that 21 CySCs survive after starvation, with four in naive state and 17 in licensed state [scenario (2), see section ‘The vSL model accurately predicts stem cell recovery dynamics after depletion’].

From the scenarios depicted above, we found a better match with scenario (2), i.e. when the population of surviving cells is mainly composed by licensed cells, with a few naive cells also surviving (Fig. 7). In this scenario, we found that all data points for the numerical experiments fall within the 95% confidence intervals of the experimental data for carrying capacities 50<*N* and ratio between division and licensing rates of 1.05<*α*<1.5 (Fig. 7B-D). When the carrying capacity becomes lower than *N*=50 the predictions of the model and the experimental results disagree, further confirming the lower boundary of *N*=50 established in the section ‘Introduction of competition for niche access yields fluctuations matching the experimental values’. Thus, our model can accurately describe the recovery dynamics of CySCs after depletion, suggesting that it has predictive value for understanding CySC dynamics beyond homeostatic conditions.

## DISCUSSION

In this work, we have investigated the role of cell states in which stem cells are licensed but not yet committed to differentiation in the maintenance of homeostasis, recovery dynamics after injury, and variability of the stem cell pool size due to randomness in stem cell division and cyst formation. We have focused on the somatic stem cells that participate in spermatogenesis in the *Drosophila* testes as a paradigmatic example. Analysis of a minimal mathematical model that considers CySCs in naive and licensed states (*SL* model) suggests that the ability of CySCs to transition back and forth between naive and licensed states, in combination with a cyst formation process involving pairs of somatic cells, provides the system with a robust homeostatic state and the ability to recover after a perturbation (see section ‘Licensing combined with pairwise differentiation of stem cells is a viable regulation strategy’).

A robust homeostatic state would also be reached if CySCs remained naive after detachment from the niche and cyst formation involved a pair of naive CySCs (leading to a system equivalent to *S*→2*S*, 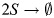). Such a scenario may not be biologically plausible in the *Drosophila* testis, as CySCs require niche signals to remain in a naive state and these signals likely do not extend into the cyst formation region, but it is instructive to consider as a hypothetical case: To achieve the same cyst formation rate as the *SL* and v*SL* models presented here, the stem cell pool size would have to be larger in a system comprising only naive CySCs. Thus, we can interpret that the role of licensing is to increase the effective stem cell pool size beyond the level that can be achieved without licensed states.

The importance of licensed states in recovery after an injury becomes evident when considering a situation in which stem cells irreversibly differentiate upon losing contact with the niche; in such a case, the system cannot recover after a perturbation. Numerical simulations further suggest that the ability of CySCs to reversibly switch between naive and licensed states is crucial to prevent stochastic extinction (see section ‘Licensing with pairwise differentiation prevents stochastic extinction and aids recovery dynamics’). However, the variability in the stem cell numbers observed experimentally is significantly lower than what would be expected from stochastic naive CySC division, licensing/de-licensing and cyst formation, which thus indicates the presence of additional regulation (see section ‘Licensing with pairwise differentiation alone cannot account for the fluctuations of the stem cell numbers’). Incorporating competition for niche access leads to the v*SL* model, which can yield lower fluctuation sizes, matching the experimentally measured values (see section ‘Introduction of competition for niche access yields fluctuations matching the experimental values’). Although competition for niche access is effective at reducing the size of fluctuations, its presence in a scenario where CySCs irreversible differentiate upon losing contact with the niche leads to stochastic extinction, typically within the lifespan of *Drosophila* (see section ‘Competition for niche access alone leads to fast extinction’). A combination of both regulation strategies is needed to reduce the variability in stem cell numbers due to the randomness of the cell division, licensing/de-licensing and cyst formation processes, while simultaneously preventing stochastic extinction of the CySC population.

It is widely accepted that stem cells require negative feedback on the cell density to maintain the correct stem cell numbers in healthy tissues (Lander et al., 2009; Jörg et al., 2021; Renardy et al., 2018; Lo et al., 2009; Kitadate et al., 2019). However, a detailed understanding of the mechanisms delivering this feedback, how they contribute to stability, to robustness against perturbations and to regeneration capacity in the stem cell pool is still lacking. Most existing mathematical models of stem cell populations that include regulation are deterministic, multi-compartment models (Lander et al., 2009; Renardy et al., 2018; Stiehl and Marciniak-Czochra, 2011; Carulli et al., 2014; Komarova, 2013; Dingli and Michor, 2006). These models are accurate for large stem cell populations. However, in stem cell niches that harbour only a handful of stem cells, stochastic effects significantly impact their population dynamics. In recent years, interesting stochastic models have explored potential regulatory mechanisms in different contexts (Greulich and Simons, 2016; Jörg et al., 2021; Corominas-Murtra et al., 2020; Kitadate et al., 2019; Hannezo et al., 2016; Vining and Mooney, 2017). Our work contributes to these efforts by exploring a regulation strategy that involves heterogeneous internal states within stem cell populations and their interplay with competition for niche access. Additionally, we provide an easily adaptable framework based on minimal mathematical models that accurately capture the stochasticity of stem cell division and differentiation processes, particularly in small population sizes.

Minimal mathematical models capture the fundamental behaviours of systems, leaving aside accessory details. Here, we assumed that naive stem cells stochastically proliferate and license, and that licensed stem cells stochastically de-license and form cysts, which provided a picture of the behaviour of the stem cell numbers in the absence of any additional mechanisms and details. However, in both the *SL* and v*SL* models, we assumed that cyst formation depends solely on the CySC population, ignoring the role of GSC availability in the cyst formation rate. This assumption is equivalent to assuming an abundance of GSCs. While this might not always be the case, we provide evidence of extensive movements of CySCs, suggesting that licensed CySCs are likely to encounter GSCs over a relatively short timescale. When introducing competition for niche access in the v*SL* model, we considered that naive CySCs compete with each other for access to niche signals, ignoring the presence of naive GSCs also competing. This consideration amounts to assuming that the naive GSC numbers remain constant throughout the process. The effect of the GSCs can be incorporated to the v*SL* model through a variable carrying capacity that is reduced after a GSC division event and increased after a GSC loses niche contact. Our modelling of competition for niche access was inspired in the birth-death process with volume exclusion model (García-Tejera et al., 2022). This model treats cells as hard spheres without any ability to deform, while CySCs have certain deformation potential. Future refinement of the v*SL* model could relax the level of volume exclusion, e.g. by allowing occasional division and de-licensing in the absence of empty spaces. The study of the combined dynamics of CySCs and GSCs, where both cell types compete for the same space within the niche, and have different deformation abilities, is a further complexification that is an interesting avenue for future research.

Stem cell populations with licensed states are present in tissues other than *Drosophila* testis. For example, licensed states have been reported in spermatogenesis in the mouse testis (Nakagawa et al., 2021; Yoshida, 2019), as well as in intestinal epithelium (e Melo and de Sauvage, 2019), hematopoietic stem and progenitor cells (Goodell et al., 2015; Laurenti and Göttgens, 2018; Haug et al., 2008), and bulge stem cells (Goodell et al., 2015). Licensing is also closely related functionally to priming in embryonic stem cells (Nichols and Smith, 2009; Ying et al., 2008; Takahashi and Yamanaka, 2016; Weinberger et al., 2016). The *SL* and v*SL* models can provide a basis for quantitative analysis in such systems. As shown in the section ‘Licensing with pairwise differentiation alone cannot account for the fluctuations of the stem cell numbers’, quantifying the variability of the stem cell numbers in homeostasis due to the randomness in the cell division processes can provide information on the regulatory mechanisms maintaining homeostasis. The models presented here can be adapted to represent other tissues with different mechanisms. Depending on the tissue of interest, the cyst formation reaction may be substituted by an alternative differentiation process, and autonomous regulation of the naive stem cells in open niches may be better modelled through competition for mitogens, rather than competition for niche access.

For example, for the intestinal epithelium, stem and transient-amplifying cell identities are thought to be strongly influenced by their position in the intestinal crypt, while stochastic exchange of positions between cells is a frequent event (Corominas-Murtra et al., 2020; Ritsma et al., 2014). Furthermore, the transition from a stem to a transit-amplifying state is achieved by the displacement of a cell to the transit-amplifying domain after the division of proximate cells. This process leads to the de-coupling of stem cell identity from cell division (Ritsma et al., 2014). Our modelling approaches incorporate both competition for niche access and state transitions that are independent of cell divisions. In our models, naive stem cells could be analogous to the stem cell population in the intestinal crypt, with the licensed cells representing the transit-amplifying cells. Moreover, transit-amplifying cells have been shown to possess the ability to dedifferentiate in regeneration (Gehart and Clevers, 2019; Chen et al., 2024), which could be considered as analogous to a de-licensing event. One difference between the intestinal crypt and our models is that the transitions from transit-amplifying to more differentiated states do not occur in pairs. This can be addressed by modifying the reaction 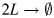 by 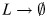. This approach could be used to explore whether transitions from TA to stem cells play a role in homeostasis, although the requirement for stable stem + TA cell pool size in individual crypts is likely far less stringent than in the *Drosophila* testes, due to the vast number of crypts. A similar approach could be used to address the impact of the tissue architecture in the robustness to perturbations and regenerative potential of the follicle stem cell niche in the *Drosophila* ovary (Kronen et al., 2014). Our study provides a useful framework to investigate how homeostasis is maintained in tissues where stem cells can adopt heterogeneous states, and to explore plausible pathways leading to regulation breakdown in disease.

## MATERIALS AND METHODS

### Measurement of CySC numbers

CySC numbers and were obtained from the datasets published by Amoyel et al. (2014). In brief, two methods of counting were used. In the first, all cells expressing Zfh1 but not Eya were counted as CySCs, giving an estimate of 44 total CySCs (including both licensed and naive populations). Based on clonal labelling, the proportion of naive stem cells has been estimated to be 14 (Amoyel et al., 2014). In the second, a membrane marker was used to determine whether Zfh1-positive cells were in contact with the niche. These were considered to represent the naive CySC population, as they are in direct contact with the niche, yielding an estimate of 18 naive stem cells (Amoyel et al., 2014). As these estimates each come with their specific inaccuracies and potential measurement errors, we considered a range of 13 to 19 naive stem cells (taking into account the standard error of measurements) when looking for agreement between model simulations and experiments.

### Deterministic modelling of stem cell population dynamics

To model the CySC population, we considered a system with two species: naive (*S*) and licensed (*L*) stem cells. In the *SL* model, naive stem cells can proliferate and license, and licensed stem cells can de-license and form cysts in pairs. This behaviour is captured by the set of reactions *S*→2*S*, 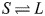 and 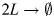. To approach the system deterministically, we considered mass-action kinetics under both well-mixed and dilute gas conditions, and took a mean field approach. If the licensing rate is larger than the division rate, the deterministic system exhibits a non-zero, stable steady state characterised by 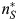 naive and 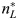 licensed stem cells, and the deterministic equations are given by (see supplementary Materials and Methods):
(1)

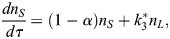

(2)

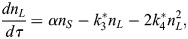
where *τ* is the time rescaled by the division rate and *α* is the ratio between the licensing and division rates.

In the case of the v*SL* model, the defining reactions are *S*+*E*→2*S*, 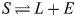 and 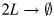, where *E* denotes the number of empty spaces within the niche. To account for limited space in the niche, we considered that the sum of naive stem cells and empty spaces remains constant, *n*_*S*_+*n*_*E*_=*N*, where *N* is the carrying capacity of the system. Taking the same assumptions as in the *SL* model, the system has a stable steady state and the deterministic equations are given by (see supplementary Materials and Methods):
(3)

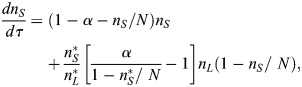

(4)

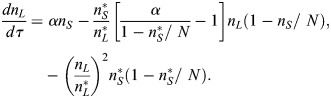
We solve Eqns 2 and 4 numerically using an adaptive Runge-Kutta (order 4) method.

### Stochastic modelling of stem cell numbers

We analysed the stochastic *SL* and v*SL* models by combining simulations and analysis. Stochastic simulations were performed using the Gillespie algorithm (Gillespie, 1976). When studying recovery dynamics (Figs 3C and 6B) we defined the recovery time as the time that takes the *in silico* tissue to reach the homeostatic number of naive CySCs for the first time after the perturbation.

To calculate the variance of the distributions of CySC numbers predicted by the *SL* model, we approximately solved the master equations of the model (see supplementary Materials and Methods). Our approximate solution makes use of the linear noise approximation (Van Kampen, 1992; Schnoerr et al., 2017; Elf and Ehrenberg, 2003), and yields analytical expressions for the variance and covariance of the naive and licensed CySC numbers:
(5)

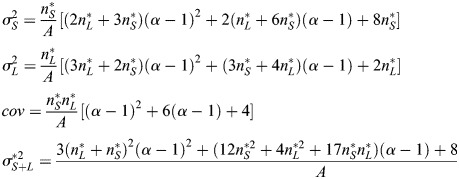
where 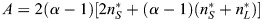. In the case of the v*SL* model, the analytical expression for the variances is rather cumbersome. Instead, we performed the linear noise approximation numerically and obtained accurate numerical estimates of the variances of the stem cell numbers according to the v*SL* model (see supplementary Materials and Methods).

### Immunohistochemistry

Testes were dissected in PBS and fixed in 4% formaldehyde for 15 min, then washed twice for 30 min each in PBS and 0.5% Triton X-100 (0.5% PBST). Samples were blocked for 1 h in PBS, 0.2% Triton X-100 and 1% BSA (PBTB) then incubated overnight at 4°C with primary antibodies diluted in PBST. These were then washed twice for 30 min each in 0.2% PBST before incubation in secondary antibodies for 2 h at room temperature, followed by two washes in 0.2% PBST and mounting on slides in Vectashield medium. We used the following primary antibodies: rabbit-anti-GFP (Invitrogen, A11122, 1:500), guinea pig anti-Zfh1 (1:4000; Wang et al., 2023 preprint), rabbit anti-Zfh1 (1:5000, a gift from R. Lehmann, Whitehead Institute, MIT, Cambridge, MA, USA). Mouse anti-Eya (eya10H6, 1:20, deposited by S. Benzer and N. M. Bonin) and mouse anti-Fas III (7G10, 1:20, deposited by C. Goodman) were obtained from the Developmental Studies Hybridoma Bank created by the NICHD of the NIH and maintained at The University of Iowa.

### Starvation and re-feeding assay

Newly eclosed (0-16 h) Oregon R males were collected and maintained at 25°C on either standard food or starvation medium, consisting of 10% sucrose, 1% agar for 14 days, with 10 flies per vial, as previously published (McLeod et al., 2010). Flies were transferred to standard food after 14 days and dissected daily for immunohistochemistry. CySCs were counted as Zfh1-positive, Eya-negative cells.

### Time-lapse imaging of explanted *Drosophila* testes

0- to 3-day old male flies of the genotype *yw hsFlp122; Fas3-GFP / Tj-Gal4; UASmodErkKTR- T2A-His2Av::mCherry* raised at 29°C after eclosion were dissected and mounted in low-melting temperature agarose and cultured with Schneider's Insect medium (Sigma, S0146) supplemented with 2.5 μl/ml human insulin (Sigma, I9278), 1% penicillin-streptomycin (Sigma, P4333) and 10% fetal bovine serum (Sigma, F2442) (Bostock et al., 2020). Confocal stacks were acquired every 10 min with a *z*-step interval of 2 μm. Movies were processed with ImageJ.

## Supplementary Material



10.1242/develop.202796_sup1 Supplementary information

Table S1. CySC counts in the starvation and re-feeding experiment. F columns indicate controls and S columns starved flies.
