# Supplementary material for: Licensing and niche competition in spermatogenesis: mathematical models suggest complementary regulation of tissue maintenance
Source: Development. 2025 Jan 2;152(1):dev202796. doi: 10.1242/dev.202796 (PMC11829763; doi:10.1242/dev.202796)
Supplement: Supplementary information [file develop-152-202796-s1.pdf]

Supplementary Materials and Methods

THE *SL* MODEL

The SL model represents a population of stem cells that can be either in naive (S) or licensed (L) state. Naive stem cells can proliferate or license stochastically, and licensed stem cells can de-license or act in pairs to form a cyst, leading to the reaction network

$$S \xrightarrow{k_1} 2S, \qquad S \underset{k_3}{\overset{k_2}{\rightleftharpoons}} L, \qquad 2L \xrightarrow{k_4} \emptyset. \tag{S1}$$

(A) Mean-field deterministic equations and stability analysis

To arrive at the deterministic equations of the SL model we assume mass-action kinetics, consider the S and L population to reside in a volume Ω as a dilute and well-mixed gas, and apply a mean-field approximation, leading to the rate equations

$$\frac{dn_S}{dt} = (k_1 - k_2)n_S + k_3 n_L, \tag{S2}$$

$$\frac{dn_L}{dt} = k_2 n_S - k_3 n_L - \frac{2k_4}{\Omega} n_L^2, \tag{S3}$$

where we assumed the approximation $n_L(n_L - 1) \approx n_L^2$. Non-dimensionalizing time $\tau = k_1 t$ and defining $\alpha = k_2/k_1$, $k_3^* = k_3/k_1$ and $k_4^* = \frac{k_4}{k_1\Omega}$ leads to

$$\frac{dn_S}{d\tau} = (1 - \alpha)n_S + k_3^* n_L, \tag{S4}$$

$$\frac{dn_L}{d\tau} = \alpha n_S - k_3^* n_L - 2k_4^* n_L^2. \tag{S5}$$

We can see that there is always a trivial steady state $[n_S^*, n_L^*] = [0, 0]$. The necessary and sufficient condition for the existence of a nontrivial steady state is that $\alpha > 1$, which means that the division rate must be lower than the licensing rate, to avoid divergence of the S population due to over-accumulation. Assuming $\alpha > 1$, the non-trivial steady state is given

Development • Supplementary information

by

$$n_S^* = \frac{k_3^{*2}}{2k_4^*(\alpha-1)^2}, \tag{S6}$$

$$n_L^* = \frac{k_3^*}{2k_4^*(\alpha-1)}. \tag{S7}$$

Note that there exists a combination of rate constants that yields any steady state value, $[n_S^*, n_L^*]$. We can re-parameterise our system according to the steady state values, observing that $k_3^* = (\alpha-1)n_S^*/n_L^*$ and $k_4^* = n_S^*/(2n_L^{*2})$, leading to

$$\frac{dn_S}{d\tau} = (\alpha-1)\left[\frac{n_S^*}{n_L^*}n_L - n_S\right], \tag{S8}$$

$$\frac{dn_L}{d\tau} = \alpha n_S - (\alpha-1)\frac{n_S^*}{n_L^*}n_L - \frac{n_S^*}{n_L^{*2}}n_L^2. \tag{S9}$$

The stability of the steady states can be analysed via the system's Jacobian

$$\mathcal{J}(n_S, n_L) = \begin{bmatrix} -(\alpha-1) & (\alpha-1)\frac{n_S^*}{n_L^*} \\ \alpha & -\frac{n_S^*}{n_L^*}\left[(\alpha-1) + \frac{2n_L}{n_L^*}\right] \end{bmatrix}. \tag{S10}$$

The Jacobian at the trivial steady state, $\mathcal{J}(0,0)$,has one positive and one negative eigenvalue, given by

$$\lambda_\pm = \frac{1}{2}(\alpha-1)(n_S^*/n_L^*)\left[-1 \pm \sqrt{1 + \frac{4\alpha(n_S^*/n_L^*)}{(\alpha-1)\left((n_S^*/n_L^*)+1\right)^2}}\right]. \tag{S11}$$

It is simple to prove that the negative eigenvalue is related to perturbations along a direction with negative slope, and the positive one to perturbations along a direction with positive slope. All real perturbations are along directions with slope higher or equal to zero so, in practice, the trivial steady state is repellent. The Jacobian in the non-trivial steady state, $\mathcal{J}(n_S^*, n_L^*)$, has eigenvalues with negative real parts, given by

$$\lambda_\pm = \frac{(\alpha-1)+(\alpha+1)n_S^*/n_L^*}{2}\left[-1 \pm \sqrt{1 - \frac{4(\alpha-1)n_S^*/n_L^*}{\left[(\alpha-1)+(\alpha+1)n_S^*/n_L^*\right]^2}}\right]. \tag{S12}$$

Both eigenvalues are real, since $\frac{4(\alpha-1)n_S^*/n_L^*}{\left[(\alpha-1)+(\alpha+1)n_S^*/n_L^*\right]^2} < \frac{2}{\alpha+1} < 1$. Hence, the nontrivial steady

Development • Supplementary information

state is always attracting, and exhibits recovery dynamics without oscillations (**Fig. S1**).

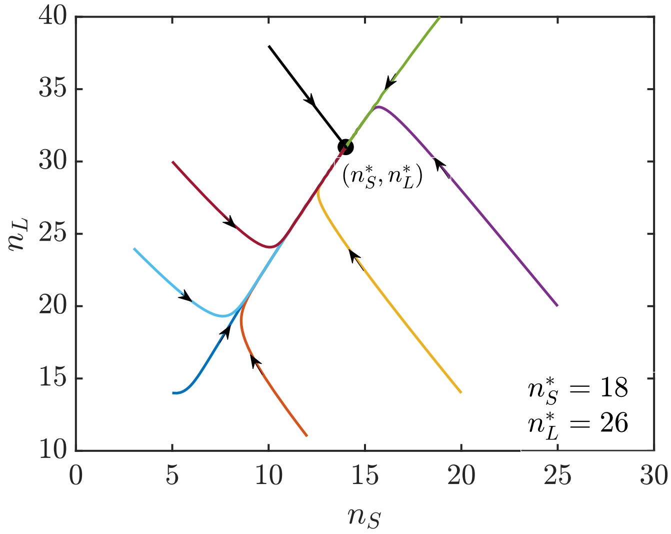

Fig. S1. Deterministic implementation of the SL model. Trajectories in the n_S , n_L configurations space for the mean-field deterministic equations of the SL model with $n^*_S = 18$, $n^*_L = 26$ and $\alpha = 3$. Regardless of the initial conditions, the system converges to $[n_S, n_L] \to [n_S^*, n_L^*]$.

(B) Stochastic implementation of the SL model

Here, we derive analytical expressions for the second moments and total distribution of the CySC numbers in homeostasis (Eq.(5)). We make use of the linear noise approximation (LNA) to the SL model's master equations (Schnoerr et al. 2017, Van Kampen 1992) and obtain expressions for the second moments of the distribution of CySC numbers in quasi-steady state, which we subsequently use to calculate the parameters of a negative binomial as an approximation for the total distribution of the stem cell numbers.

The Jacobian at the nontrivial steady state of the mean-field deterministic equations is given by evaluating the jacobian $\mathcal{J}$ given by Eq. S10 at $(n_S, n_L) = (n_S^*, n_L^*)$:

$$\mathcal{J}(n_S^*, n_L^*) = \begin{bmatrix} -(\alpha-1) & (\alpha-1)\frac{n_S^*}{n_L^*} \\ \alpha & -\frac{n_S^*}{n_L^*}\left[(\alpha+1)\right] \end{bmatrix}, \tag{S13}$$

where n_S^* and n_L^* are the homeostatic numbers of naive and licensed CySCs respectively, and α is the ratio between the licensing and division rates of naive CySCs. The linear

Development • Supplementary information

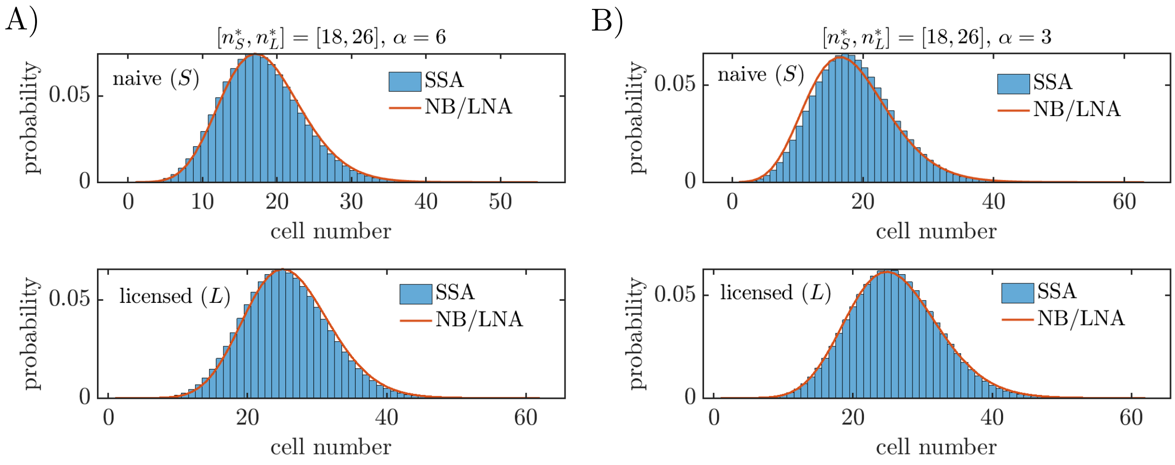

FIG. S2. Negative binomial/LNA approximation (red line) to the solution of the Kolmogorov's forward equation for the SP model in steady state (Eq. (S18)), for the experimentally estimated values of n_S^* and n_L^*, for $\alpha = 6$ (**A**) and $\alpha = 3$ (**B**). Histograms correspond to stochastic simulations (extinction events are excluded).

noise approximation amounts to performing the van Kampen's system-size expansion of the master equation in powers of $N^{-1/2}$ and collecting the terms of order N^0, obtaining a Fokker-Planck equation (Van Kampen 1992). For reaction networks consisting of many species (here naive and licensed CySCs), it is convenient to adopt a matrix formulation for the LNA, as introduced in (Elf and Ehrenberg 2003). Under such formulation, the variances and covariances between species in steady state are given by the solution to the Lyapunov equation

$$\mathcal{J}\mathbf{C} + \mathbf{C}\mathcal{J}^{\mathrm{T}} + \mathbf{\Omega}\mathbf{S}\,diag(\mathbf{f}(\phi))\mathbf{S}^{\mathrm{t}} = \mathbf{0}, \tag{S14}$$

where $\mathcal{J}$ is the Jacobian of the deterministic equations in steady state, $\mathbf{C}$ is the variance matrix to be calculated, Ω is the system volume (here taken as $\Omega = 1$ without losing generality), $\mathbf{S}$ is the stoichiometric matrix and $\mathbf{f}$ the propensity vector. The stoichiometric matrix of the SL model's reaction network is given by

$$S = \begin{bmatrix} 1 & -1 & 1 & 0 \\ 0 & 1 & -1 & -2 \end{bmatrix}, \tag{S15}$$

where the first row represents the net changes of S species and L is encoded in the second

Development • Supplementary information

row. Considering mass-action kinetics, the propensity vector in steady state is given by

$$\mathbf{f} = \left[n_S^*, \alpha n_S^*, n_S^*(\alpha-1), \frac{n_S^*}{2n_L^*}(n_L^*-1)/\Omega \right]. \tag{S16}$$

Solving Lyapunov's equation for the system Jacobian and diffusion matrix yields,

$$\begin{aligned}
\sigma_S^2 =& \frac{n_S^*}{2(\alpha-1)}\left[\frac{(2n_L^*+3n_S^*)(\alpha-1)^2+2(n_L^*+6n_S^*)(\alpha-1)+8n_S^*}{2n_S^*+(\alpha-1)(n_S^*+n_L^*)}\right],\\
\sigma_L^2 =& \frac{n_L^*}{2(\alpha-1)}\left[\frac{(3n_L^*+2n_S^*)(\alpha-1)^2+(3n_S^*+4n_L^*)(\alpha-1)+2n_L^*}{2n_S^*+(\alpha-1)(n_S^*+n_L^*)}\right],\\
cov =& \frac{n_S^* n_L^*}{2(\alpha-1)}\left[\frac{(\alpha-1)^2+6(\alpha-1)+4}{2n_S^*+(\alpha-1)(n_S^*+n_L^*)}\right],\\
\sigma_{S+L}^{*2} =& \frac{3(n_L^*+n_S^*)^2(\alpha-1)^2+(12n_S^{*2}+4n_L^{*2}+17n_S^*n_L^*)(\alpha-1)+8n_S^*(n_S^*+n_L^*)+2n_L^{*2}}{2(\alpha-1)\left[(\alpha-1)(n_S^*+n_L^*)+2n_S^*\right]}.
\end{aligned} \tag{S17}$$

Note that when $\alpha \to 1$ the fluctuation sizes tend to ∞.

Instead of taking the Gaussian approximation yielded by the LNA, we use the expressions for the first two moments of the distributions to calculate the parameters of negative binomials for the probability distributions of the number of naive and licensed CySCs in homeostasis. In contrast to the LNA, a negative binomial is able to capture the skewness of the distributions. The resulting (quasi) steady state distributions for the S and L species read

$$\begin{aligned}
P_S(k) &= \frac{(k+r_S+1)!}{(r_S-1)!k!}(1-q_S)^k q_S^{r_S},\\
P_L(k) &= \frac{(k+r_L+1)!}{(r_L-1)!k!}(1-q_L)^k q_L^{r_L},
\end{aligned} \tag{S18}$$

where $r_S = n_S^{*2}/(\sigma_S^2 - n_S^*)$, $q_S = n_S^*/\sigma_S^2$, $r_L = n_L^{*2}/(\sigma_L^2 - n_L^*)$ and $q_L = n_L^*/\sigma_L^2$. The negative binomial/LNA approximation is in good agreement with stochastic simulations for most values of α (Fig. S2 A and B). For lower values of α extinction events are more frequent. As a consequence, the probability distribution of the surviving trajectories (captured by the SSA) is biased towards higher stem cell numbers, which creates a mismatch between the first moment of the distributions calculated via the SSA and the NB/LNA approximation (see Fig. S2 B). The second moment, however, remains accurate for most values of α, only showing disagreement for $\alpha \lesssim 1.1$ (since the LNA prediction diverges when $\alpha \to 1$), as shown in Fig. S3).

Development • Supplementary information

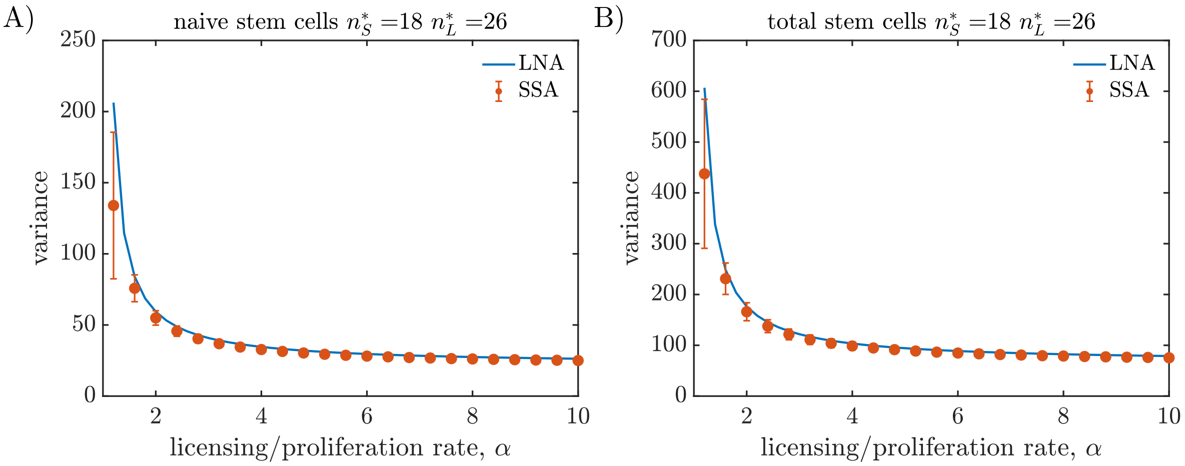

FIG. S3. Second moments of the distribution of naive (**A**) and total (**B**) stem cell numbers in quasi-steady state, as a function of α for the SL model. The variance measurements (orange dots) are averaged over 5×10^3 realisations of the SSA with 1×10^4 time points. Error bars show one standard deviation in the ensemble of realisations. The LNA predictions (blue lines), obtained from Eq. (S17), are in agreement with the SSA measurements.

ANALYSIS OF ALTERNATIVE SCENARIO TO THE SL MODEL

In section II B of the main manuscript we analyse an alternative scenario in which naive stem cells irreversibly differentiate when they lose contact with the niche, which can be captured by the reaction network:

$$S \xrightarrow{\mu_1} 2S, \qquad S \xrightarrow{\mu_2} L, \qquad 2L \xrightarrow{\mu_3} \emptyset. \tag{S19}$$

The mean-field deterministic equations of the system are

$$\begin{aligned} \frac{dn_S}{d\tau} =&(1-\alpha)n_S, \\ \frac{dn_L}{d\tau} =&\alpha n_S - 2\gamma n_L^2, \end{aligned} \tag{S20}$$

where $\alpha = \mu_2/\mu_1$, $\gamma = \mu_3/(\mu_1\Omega)$, being Ω the system volume, and $\tau = \mu_1 t$. For the mean-field equations to have a steady state $[n^*{}_S, n^*{}_L]$ it must be $\alpha = 1$ and $\gamma = n^*{}_S/(2n^*{}_L{}^2)$. It is simple to prove that the system Jacobian in steady state has eigenvalues $\lambda_1 = 0$ and $\lambda_2 = -2n^*{}_S/n^*{}_L$, the null eigenvalue implying that the system would not go back to the original steady state after perturbation, as all the points in the curve $n_S = 2\gamma n^2{}_L$ are steady states.

Development • Supplementary information

THE VSL MODEL

The vSL model can be seen as an extension of the SL model that incorporates competition for niche access in the naive stem cell population, modelled as a volume exclusion effect (García-Tejera et al. 2022). Naive stem cells (S) can proliferate stochastically, provided that they find empty spaces within the niche (E). They can also license by moving away from the niche, thus leaving an empty space available. Licensed stem cells can de-license by taking up an empty space in the niche, or act in pairs to form a cyst, leading to the reaction network

$$S + E \xrightarrow{k_1} 2S, \qquad S \underset{k_3}{\overset{k_2}{\rightleftharpoons}} L + E, \qquad 2L \xrightarrow{k_4} \emptyset. \tag{S21}$$

To account for a finite space in the niche, we introduce a carrying capacity N by demanding that at all times the number of naive stem cells plus empty spaces remains constant, i.e., $n_S + n_E = N$.

(A) Mean-field deterministic equations and stability analysis

Assuming mass-action kinetics, considering the well-mixing and dilute gas hypotheses, and imposing $n_E = N - n_S$, where N is the niche's carrying capacity leads to the mean-field equations

$$\begin{aligned} \frac{dn_S}{d\tau} &= n_S(1 - n_S/N) - \frac{k_2}{k_1} n_S + \frac{k_3}{k_1} n_L(1 - n_S/N), \\ \frac{dn_L}{d\tau} &= \frac{k_2}{k_1} n_S - \frac{k_3}{k_1} n_L(1 - n_S/N) - \frac{2k_4}{k_1 N} n_L(n_L - 1), \end{aligned} \tag{S22}$$

where we have defined the non-dimensional time $\tau = k_1 t$. Let us now assume the existence of a nontrivial steady state characterised by $n_S = n_S^* \leq N$ and $n_L = n_L^*$, where both $n_S^*, n_L^* \neq 0$. We prove the existence of such a steady state later on. Substitution of the steady state values in the rate equations leads to

$$\frac{k_3}{k_1} = \frac{n_S^*}{n_L^*}\left[\frac{\alpha}{1 - n_S^*/N} - 1\right], \qquad \frac{2k_4}{Nk_1} = \frac{n_S^*(1 - n_S^*/N)}{n_L^*(n_L^* - 1)}, \tag{S23}$$

where we have defined $\alpha = k_2/k_1$. Substitution of the identities (S23) in the rate equations (S22) leads to

$$\begin{aligned}\frac{dn_S}{d\tau} =&(1-\alpha-n_S/N)n_S + \frac{n_S^*}{n_L^*}\left[\frac{\alpha}{1-n_S^*/N}-1\right]n_L(1-n_S/N),\\ \frac{dn_L}{d\tau} =&\alpha n_S - \frac{n_S^*}{n_L^*}\left[\frac{\alpha}{1-n_S^*/N}-1\right]n_L(1-n_S/N) - \frac{n_S^*(1-n_S^*/N)}{n_L^*(n_L^*-1)}n_L(n_L-1),\end{aligned} \tag{S24}$$

which is Eq.(4) of the main text.

To prove that a nontrivial steady state (n_S^*, n_L^*) exists consider the nullclines for the rate equations (S22), given by

$$\begin{aligned}n_L = f_1(n_S) =&\frac{k_1}{k_3}n_S\left(\frac{\alpha}{1-n_S/N}-1\right),\\ n_L = f_2(n_S) =&\frac{k_3N}{4k_4}\sqrt{\left(1-\frac{n_S}{N}\right)^2+\frac{8k_4k2n_S}{Nk_3^2}},\end{aligned} \tag{S25}$$

as shown in Fig. S4. The existence of the nontrivial steady state with $n_S^* \leq N$ is a consequence of the following conditions, which are fulfilled for $\alpha > 1$: a) $f_1(0) = f_2(0) = 0$, b) $\partial f_1/\partial n_S(0) > 0$, c) $\partial f_1/\partial n_S \to \infty$ as $n_S \to N^-$, d) $\partial^2 f_1/\partial n_S^2 > 0$ in $[0, N)$, e) $\partial f_2/\partial n_S(0) > \partial f_1/\partial n_S(0)$, and f) $\partial^2 f_2/\partial n_S^2 < 0$ in $[0, N)$.

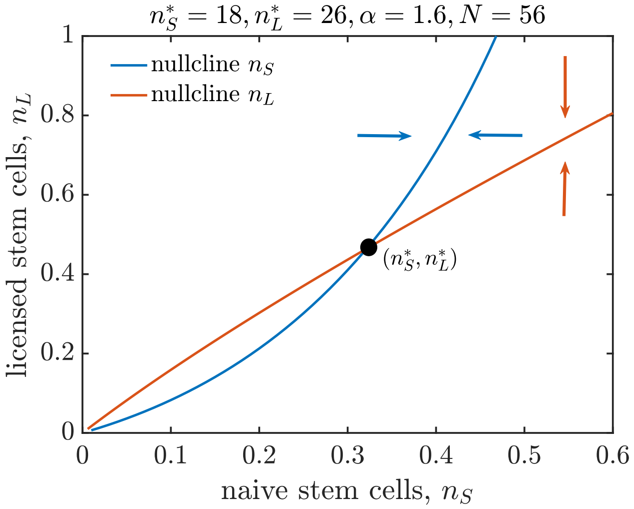

Fig. S4. Phase portrait of the vSL model. The steady states ((0, 0) and $(n^*{}_S, n^*{}_L)$) are the intersection points of the nullclines (zero-growth curves) of n_S (blue) and n_L (orange). Arrows represent the directions of growth outside the nullclines, displaying (n_S^*, n_L^*) as a stable steady state.

Development • Supplementary information

Let $\mathbf{f}(n_S, n_L) = [f_1(n_S, n_L), f_2(n_S, n_L)]$ with $f_1 = (1-\alpha-n_S/N)n_S + \frac{n_S^*}{n_L^*}\left[\frac{\alpha}{1-n_S^*/N} - 1\right] n_L(1-n_S/N)$ and $f_2 = \alpha n_S - \frac{n_S^*}{n_L^*}\left[\frac{\alpha}{1-n_S^*/N} - 1\right] n_L(1-n_S/N) - \frac{n_S^*(1-n_S^*/N)}{n_L^*(n_L^*-1)} n_L(n_L-1)$. Defining $\phi_S = n_S/N$ and $\phi_L = n_L/N$, the Jacobian of $\mathbf{f}$ at the steady state is given by

$$J_{\mathbf{f}}(\phi_S, \phi_L) = \begin{bmatrix} 1-\alpha-\phi_S^*\left(\frac{\alpha}{1-\phi_S^*}+1\right) & \frac{\phi_S^*}{\phi_L^*}(\alpha-1+\phi_S^*) \\ \alpha+\phi_S^*\left(\frac{\alpha}{1-\phi_S^*}-1\right) & -\frac{\phi_S^*}{\phi_L^*}(\alpha+1) \end{bmatrix}, \tag{S26}$$

where we have approximated $n_L(n_L-1) \approx n_L^2$. Eigenvalue calculation yields two real, negative eigenvalues for $\alpha > 1$, which proves the linear stability of the nontrivial steady state. The stability of the steady state also becomes evident upon inspection of the phase portrait of the vSL model in Fig. S4.

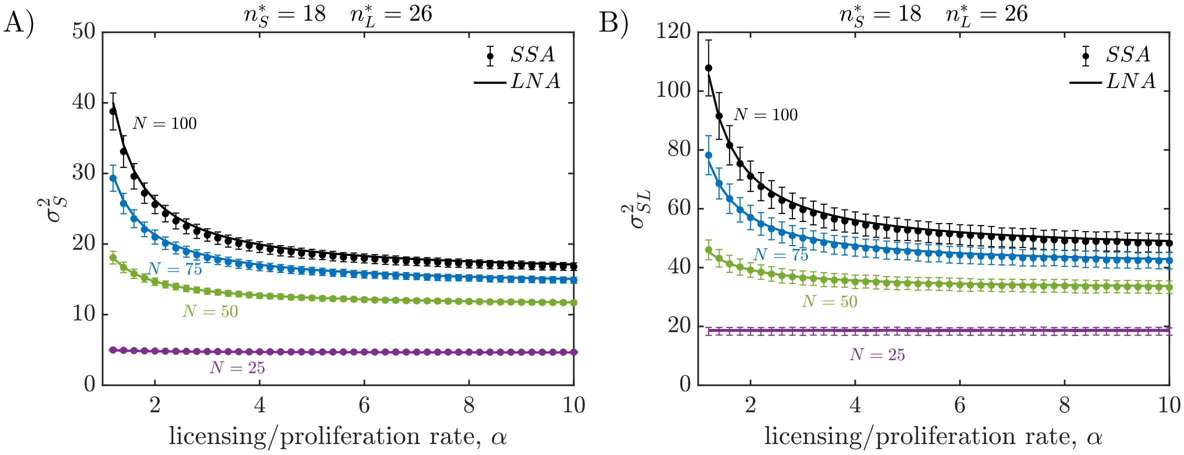

Fig. S5. Second moments of the distribution of naive (**A**) and total (**B**) stem cell numbers in quasi-steady state, as a function of α for the vSL model with different carrying capacities N. The variance measurements (dots) are averaged over 1×10^3 realisations of the SSA with 1×10^4 time points. Error bars show one standard deviation in the ensemble of realisations. The LNA predictions (lines), obtained from numerically solving the Lyapunov equation (Eq. (S14)), are in agreement with the SSA measurements.

(B) Stochastic implementation of the vSL model

We follow a similar procedure than for the SL model in Sec. SI B, with the constraint that for the vSL model analytical solutions of the Lyapunov equation (Eq. (S14)) are rather cumbersome. Instead, we solve the Lyapunov equation numerically for each parameter set

Development • Supplementary information

to find the second moments of the distributions of stem cell numbers in quasi-steady state. The second moments of the distribution in quasi-steady state obtained via the LNA are in good agreement with SSA calculations (Fig. S5).

FURTHER EVIDENCE THAT DE-LICENSING IS A FREQUENT EVENT

We performed an additional experiment to provide further evidence showing that de-licensing events through movement of cells from the second to the first row is a frequent event. This experiment aims to trace the lineage of licensed cells by performing *spict* lineage tracing and using the fact the regions of high Tor activity and *spict* expression are correlated. We induce GFP in licensed cells and measure the presence of the label in the first row at subsequent times (Fig. S6).

We used *spict-Gal4* which previous work has shown is expressed in CySCs located two cell diamaters from the hub (Chiang et al. 2017), combined with a temperature-sensitive Gal80 (McGuire et al. 2004) to lineage trace *spict*-expressing cells over time. Male flies of the genotype *y,w,hs-flp122; spict-Gal4/ Act>stop>Gal4, UAS-GFP; Tub-Gal80ts/UAS-flp*; were raised at 18°C. Newly eclosed males (0-2 days) were shifted to 29°C to inactivate Gal80 and dissected at 6, 12, 18, 24, and 48 hours post temperature shift.

A limitation of this experimental approach is that we cannot rule out that a small fraction of CySCs that contact the hub also undergo recombination induced by Spict-Gal4-driven Flippase, either due to leakage of the Gal80 system or due to occasional real expression of Spict-Gal4 in first-row cells. Nevertheless, the experiment, together with other data, supports the idea that cells in the second row do contribute to the first row at a non-negligible rate. This is further supported by the observation that mitosis only occurs in CySCs in contact with the hub (Cheng et al. 2011), while DNA replication is seen in cells in both the first and second rows (de la Maza et al. 2022), which implies that cells that replicate their DNA in the second row return to the first row to undergo mitosis.

Development • Supplementary information

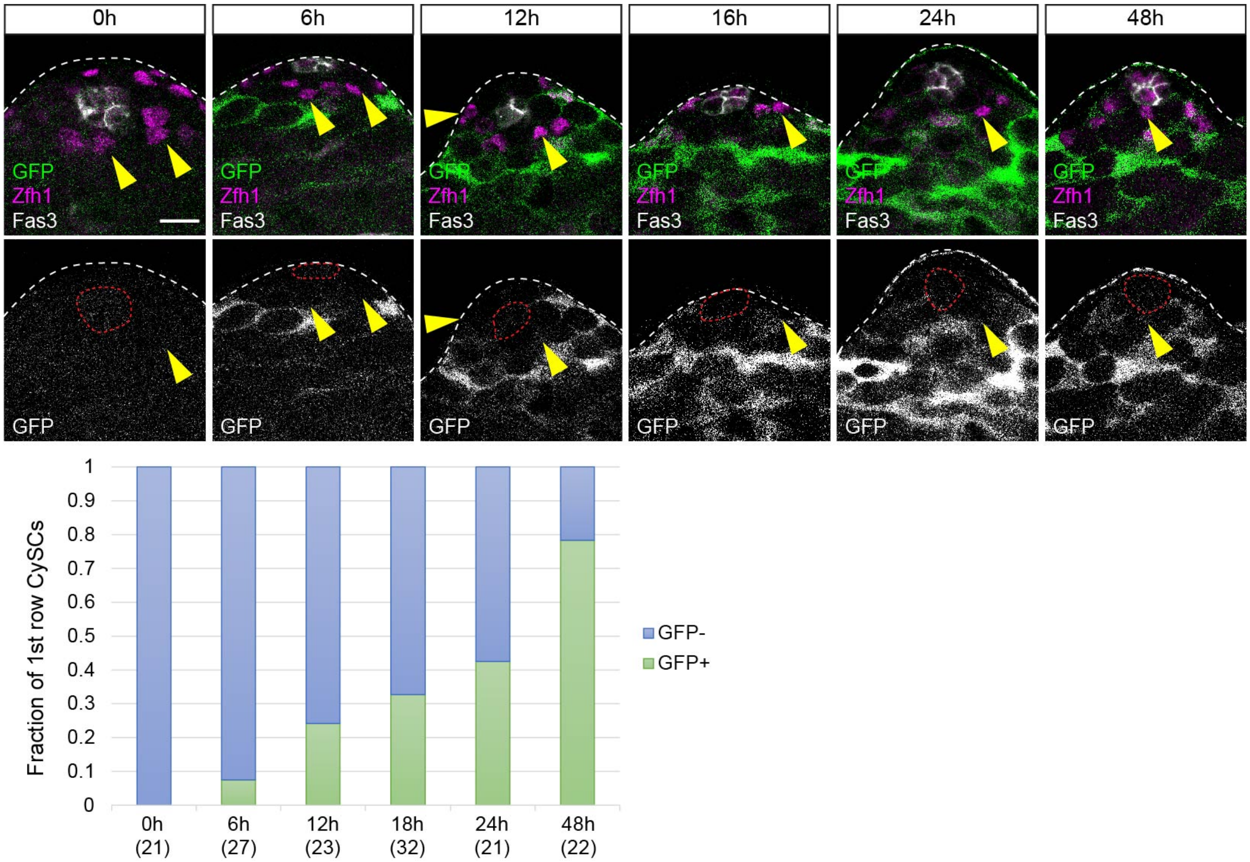

Fig. S6. Lineage tracing shows frequent movement of CySCs from the second row to the first. Top: Images from testes in which spict-Gal4 expressing cells were permanently labelled with GFP (green and single channel below). The time of temperature shift to initiate the lineage trace is indicated, no cells were labelled at the initial time point (0h), while at 6h, only 2nd row cells are labelled. Over time, more and more cells in the first row were labelled. CySCs were labelled with antibodies against Zfh1 (magenta), Arrowheads indicate unlabelled 1st row CySCs, identified for their proximity to the niche, labelled with antibodies against Fas3 (white, outlined with a red dotted line). Scale bar: 10 μm. Bottom: quantification of the fraction of GFP-positive or negative CySCs in the first row at each time point. The number in parentheses indicates the number of samples examined.

Table S1. CySC counts in the starvation and re-feeding experiment. F columns indicate controls and S columns starved flies.

Available for download at
https://journals.biologists.com/dev/article-lookup/doi/10.1242/dev.202796#supplementary-data

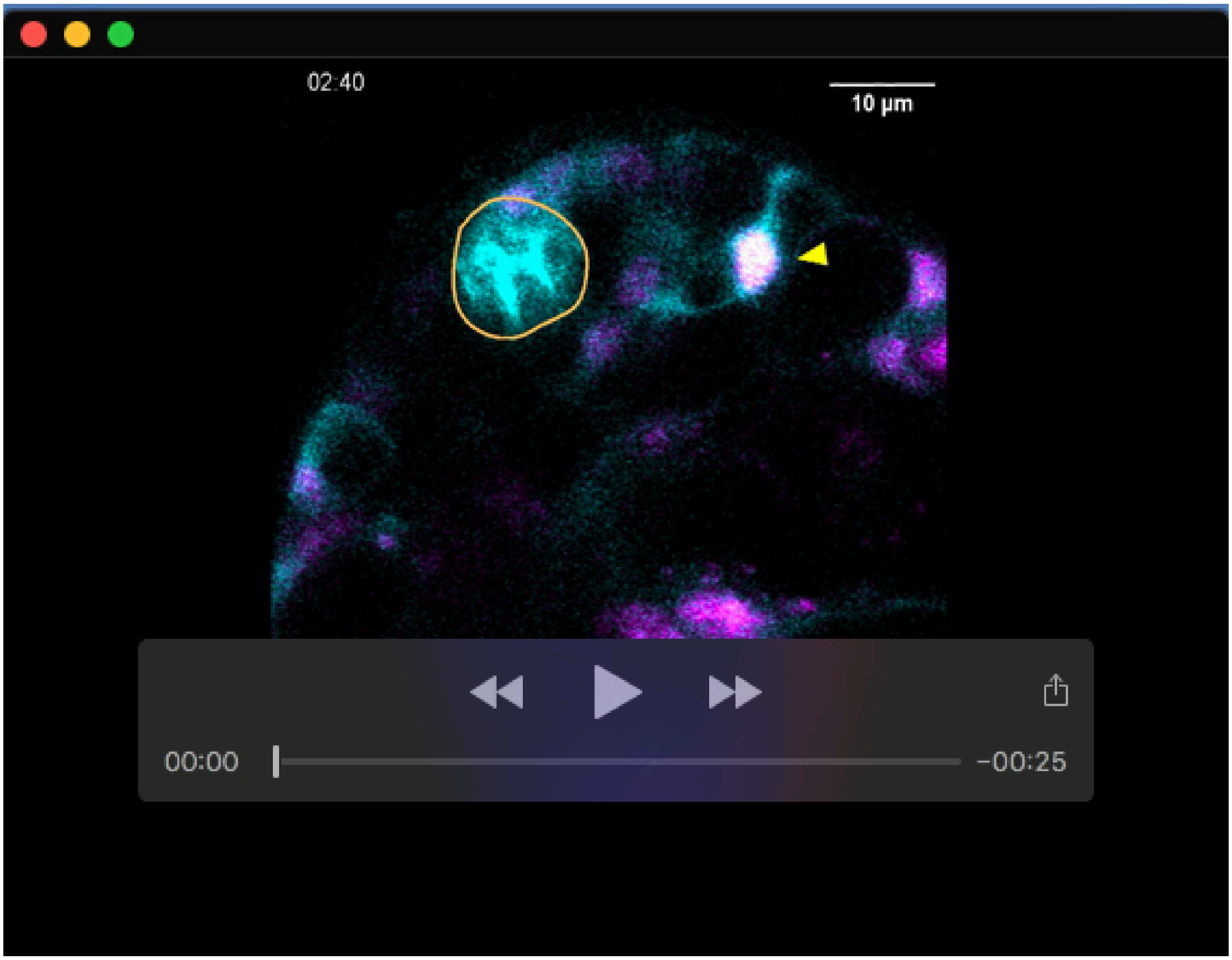

Movie 1. CySCs move extensively around the niche. Time-lapse movie highlighting a CySC (arrowhead) that moves laterally around the niche. CySCs are labelled with GFP (cyan) and nuclear RFP (magenta). The niche is labelled with Fas3-GFP (cyan, bright), but in many frames, the niche is in an adjacent Z-plane and its rough position is outlined with a yellow line. The highlighted CySC contacts several different germ cells (unlabelled) over the course of the movie. Scale bar: 10 μm.

J. Cheng, A. Tiyaboonchai, Y. M. Yamashita, and A. J. Hunt. Asymmetric division of cyst stem cells in drosophila testis is ensured by anaphase spindle repositioning. *Development*, 138(5):831– 837, 2011.

A. C.-Y. Chiang, H. Yang, and Y. M. Yamashita. spict, a cyst cell-specific gene, regulates starvation-induced spermatogonial cell death in the drosophila testis. *Scientific reports*, 7(1):40245, 2017.

D. S. de la Maza, S. Hof-Michel, L. Phillimore, C. Bökel, and M. Amoyel. Cell-cycle exit and stem cell differentiation are coupled through regulation of mitochondrial activity in the drosophila testis. *Cell Reports*, 39(6), 2022.

J. Elf and M. Ehrenberg. Fast evaluation of fluctuations in biochemical networks with the linear noise approximation. *Genome research*, 13(11):2475–2484, 2003.

R. García-Tejera, L. Schumacher, and R. Grima. Regulation of stem cell dynamics through volume exclusion. *Proceedings of the Royal Society A*, 478(2266):20220376, 2022.

R. E. McGuire, Z. Mao, and R. L. Davis. Spatiotemporal gene expression targeting with the target and gene-switch systems in drosophila. *Science's STKE*, 2004(220):pl6–pl6, 2004.

D. Schnoerr, G. Sanguinetti, and R. Grima. Approximation and inference methods for stochastic biochemical kinetics—a tutorial review. *Journal of Physics A: Mathematical and Theoretical*, 50 (9):093001, 2017.

N. G. Van Kampen. *Stochastic processes in physics and chemistry*, volume 1. Elsevier, 1992.

Development • Supplementary information
