# Supplementary material for: Licensing and niche competition in spermatogenesis: mathematical models suggest complementary regulation of tissue maintenance
Source: Development. 2025 Jan 2;152(1):dev202796. doi: 10.1242/dev.202796 (PMC11829763; doi:10.1242/dev.202796)
Supplement: Supplementary file 1 [file develop-152-202796_review_history.pdf]

Licensing and niche competition in spermatogenesis: mathematical models suggest complementary regulation of tissue maintenance

Rodrigo García-Tejera, Jing-Yi Tian, Marc Amoyel, Ramon Grima and Linus J. Schumacher
DOI: 10.1242/dev.202796

Editor: Paul Francois

Review timeline
Original submission: 16 February 2024
Editorial decision: 11 April 2024
First revision received: 20 September 2024
Editorial decision: 8 October 2024
Second revision received: 31 October 2024
Accepted: 21 November 2024

Original submission

First decision letter

MS ID#: DEVELOP/2024/202796

MS TITLE: Licensing and competition of stem cells at the niche combine to regulate tissue maintenance

AUTHORS: Rodrigo Garcia-Tejera, Marc Amoyel, Ramon Grima, and Linus Schumacher

I have now received all the referees' reports on the above manuscript, and have reached a decision. The referees' comments are appended below, or you can access them online: please go to BenchPress and click on the 'Manuscripts with Decisions' queue in the Author Area.

As you will see, the referees express considerable interest in your work, but have some significant criticisms and recommend a substantial revision of your manuscript before we can consider publication. If you are able to revise the manuscript along the lines suggested, which may involve further experiments, I will be happy receive a revised version of the manuscript. Your revised paper will be re-reviewed by one or more of the original referees, and acceptance of your manuscript will depend on your addressing satisfactorily the reviewers' major concerns. Please also note that Development will normally permit only one round of major revision. If it would be helpful, you are welcome to contact us to discuss your revision in greater detail. Please send us a point-by-point response indicating your plans for addressing the referees' comments, and we will look over this and provide further guidance.

Please attend to all of the reviewers' comments and ensure that you clearly highlight all changes made in the revised manuscript. Please avoid using 'Tracked changes' in Word files as these are lost in PDF conversion. I should be grateful if you would also provide a point-by-point response detailing how you have dealt with the points raised by the reviewers in the 'Response to Reviewers' box. If you do not agree with any of their criticisms or suggestions please explain clearly why this is so.

Reviewer 1

Advance summary and potential significance to field

© 2025. Published by The Company of Biologists under the terms of the Creative Commons Attribution License (https://creativecommons.org/licenses/by/4.0/).

The work presents a model for differentiation of cyst stem cells in Drosophila spermatogenesis. This model includes the phenomenon of "licensing", for which experimental evidence has been provided in the past.
Licensing means that stem cells become primed for differentiation, attaining a state "licensed" for differentiation, from which they may commit to terminal differentiation, but they also retain the potential to return to the (naive) stem cell state, also called de-licensing. One particular additional constraint is that cyst stem cells can only commit to differentiation in pairs, by simultaneously engulfing a germline stem cells in a cyst. The authors study a stochastic version of the model, backed up by deterministic equations for mean values in the Supplemental Information. They notice a certain degree of robustness to perturbations in the model, which is interpreted as a stable homeostatic state, yet stochastic fluctuations can lead to extinction of the stem cell population in some cases. The authors claim that the stability of homeostasis is the result of (de-)licensing. Through addition of competition for niche space, realistic rates of extinction and fluctuation variances, that match experimental data, can be achieved.

The manuscript is well written and the modelling results seem robust and correct. The model is well motivated by experimental observations and is likely capturing the relevant features of the real world scenario. When spatial competition is taken into account, its predictions can be brought in agreement with experimental data, which further confirms the model assumptions. The importance of this work is that the model incorporates mechanisms to confer stable homeostasis in spermatogenesis (and possibly other systems). However, I am convinced that the authors misinterpreted the particular mechanism within the model which is responsible for maintaining homeostasis. Regarding this, see my further comments below.

Comments for the author

I do have doubts about the claim that homeostasis (as in, a stable steady state of expected cell numbers) is the result of (de-)licensing. I am rather convinced that the key ingredient to confer stable homeostasis is the fact that commitment to differentiation occurs through pairwise interaction between cells. In fact, (de-)licensing is not required per se for that. This can be seen when analysing the deterministic equation for the mean cell numbers, Eqs. (9,10) in the Supplemental Information. If differentiation of licensed cells would not occur pairwise, the last term of Eq. (10) were linear, and in fact the whole system were linear. In that case, no non-zero asymptotically stable state can exist, only possibly a neutrally stable state which features diverging fluctuations over time. In addition to diverging fluctuations, such a neutrally stable state could only be achieved through accurate fine-tuning of proliferation vs. differentiation rates. Thus, the pairwise interaction is necessary for stable homeostasis. The authors claim that without (de-)licensing homeostasis does not prevail. However, this is only because without (de-)licensing, commitment to terminal differentiation, in that case the transition from stem cells to L-cells (which are not "licensed" anymore in that scenario), occurs before the pairwise differentiation. If instead, in that scenario, commitment would be through pairwise interaction, that is, involving two naive stem cells, then we would have again a stable homeostatic state. This can be seen in Eq. (25) of the Supplemental Information, when we would have dn_S/dtau = n_s - alpha*n_s^2, which yields a stable steady state for alpha>1.

Hence, the crucial mechanism to enable stable homeostasis is that, at the point were cells irreversibly commit to terminal differentiation, they do so via pairwise interaction (any other non-linear interaction could work as well), irrespective of whether (de-)licensing occurs or not. (De-)Licensing affects this only in so far, as it moves the point of irreversible commitment further down the hierarchy. I therefore think that the claim that stable homeostasis is caused by (de-)licensing cannot be upheld, and that the authors should adapt their main claim and to consider more precisely the effect of the pairwise interaction on homeostatic control. This would likely also require to change the title, which includes the claim.

The competition for finite niche space, on the other hand, is a further viable mechanism to maintain a stable homeostatic state, and to reduce fluctuations in cell numbers.

© 2025. Published by The Company of Biologists under the terms of the Creative Commons Attribution License (https://creativecommons.org/licenses/by/4.0/).

Beyond this concern, I have only one further suggestion: it would be good to see the results for the vSL model in a figure, analogue to Fig. 3 for the SL-model. Currently, the results of the vSL model are less clear without visualisation.

Reviewer 2

Advance summary and potential significance to field

A mathematical model is developed for a community of stem cells with two different states, named naiive and primed, which can exchange. Additionally, only primed cells can directly differentiate. The model is mostly potentially general but is being applied to the Drosophila testis and therefore has a specialized component that differentiation of primed cells only occurs in pairs (which must be spatially close and in contact with a germline gonialblast, also ready to differentiate). The effect of dividing stem cells into two states was compared to a single state in the context of no other constraints or when an additional feature, competition for niche access, is introduced by modeling using some experimental measurements of average populations of the two stem cell states. The reported results are that the extra state reduces modeled fluctuations in stem cell numbers (but not sufficiently to reflect experimental observations) and supplements the effect of niche competition alone, so that extinctions of the stem cell population are less frequent and compatible with observations.

Comments for the author

This reviewer has paid some attention to modeling but does not have expertise to evaluate the modeling details per se or to compare in a detailed way to other relevant modeling studies. My perception is that the use of mathematical models might have three major components- (i) formulation of the model according to sufficient (never complete) experimental knowledge of key parameters, (ii) assumptions (mostly simplifying to assume certain factors are constant, that others may not be limiting, or that there are discrete states rather than a continuum or multiplicity of states) that allow creation of a relatively simple model to examine predicted or simulated cell behavior, and ideally (iii) making an alteration to the experimental system that tests a prediction of the model or shows its benefit.

I have some significant reservations in all three areas.
Most straightforward, there is no testing of the model by making one or more specific experimental perturbations. For me, this is a serious deficiency. No model is going to be truly accurate, but why should anyone take special note of this model if it has not proven its value in this way. The modeled system affords many genetic tests. If it is possible to test the model well, surely this is the place to report that.

The connection between experimental observations and the formulation of the model needs some further clarification.
The model appears to be framed according to which cells contact the niche. I believe that translates to primed cells being “two diameters” from the niche and not in contact. This should be confirmed. A potentially important point here is whether this simple duality appears to exist in reality. Is there not evidence for different degrees of contact and for different expression of reporters of signals deriving from the hub niche (and a ”differentiation niche”) for a given nuclear location (1 vs 2 cell diameters away)?
The model includes cells 2 diameters away sometimes moving back to contact the hub. The model seems mainly built on the basisof reference [5], wherein Amoyel et al, 2014 is used to support the statement that “CySC replacement by neighbors is a frequent event”, then noting that CySCs are identified by contact with the niche. This is not equivalent to evidence of cells moving from “layer 2” to “layer 1” and there are no such data in the 2014 paper.
While there is prior evidence that JAK-STAT and some other signals are higher in cells next to the niche, while the PI3K pathway is higher in more distant cells, and that the former sets of signals affect division rate, while the latter contributes to final differentiation, there seem to be numerous uncertainties also (as would be expected). For example, do any of these signals also affect the movement of cells from one layer to another? In other words, are the circumstances (signals,

© 2025. Published by The Company of Biologists under the terms of the Creative Commons Attribution License (https://creativecommons.org/licenses/by/4.0/).

geometry) often affecting more than one model parameter? If so, can each parameter really be considered independent?
It seems particularly difficult to exclude geometry. First, the potential for final differentiation depends on the availability of a neighboring "licensed" cell and a suitable germline cell. Both of these emerged locally from stem cells that were competing for niche access. Second, the likelihood of a cell returning to contract the hub depends on available space. This will depend on the state of various nearby cells (division of cells at the niche, attempted return of neighbors). Third, transition of S L could similarly depend on the density of niche-adjacent cells.
In the end, a model introduces competition for the niche as an additional factor but my understanding is that this does not negate these concerns about independent parameters of the basic "SL" model.

Regarding the model itself,
1. I find it is laid out understandably for the non-expert. I am interested in the authors' opinion on some other models (tangential to the main topic), which consider the frequency of outcomes of cell divisions (producing two stem cells, two non-stem cells or one of each) even when the paradigm does or may actually involve separate division and differentiation events. I have always thought that is inherently flawed and am glad to see the formulation presented here is different.
2. The assumption that germline cell status is not relevant seems fragile- germline status might be instructive and limiting. The omission of spatial relationship between two potentially differentiating cells, I think, effectively assumes that an average separation will be uniform, which seems very unlikely.
3. It seems that there could just as easily be several states, rather than just S and L. I understand that simplification and seeing what just two states can accomplish has merits, but this leaves open the question of what might be added by multiple states.
4. The chosen method for adding niche competition is presumably not the only modeling choice, nor is it necessarily true that niche competition is really a single process (for example, since signal strengths may play complicated roles, competition for a certain space with niche contact may not be the only relevant, operative parameter). I am therefore suspicious of whether the S/L division is just one of several possible arrangements (compatible with reality) that complement and add to properties endowed by niche competition. Specifically, the niche competition method talks about empty space or not, as black or white, rather than degrees of space available. It would help to clarify whether introducing a variable space is likely in reality or practical in a model.
5. I think it would be very useful to compare the authors' model with various other published models dealing with the same issues of stabilizing stem cell populations. It seems to me there is considerable overlap- hence, it is appropriate to have expert comment.
6. It is a detraction that the model is tailored to one paradigm. How would the model differ if applied, for example, to the mouse small intestinal crypt?

Finally, I would say that it seems intuitively obvious that having an intermediate state with reversible exchanges will improve reliability. One way is simply by increasing the total population of stem cells. It may be useful to compare the S/L dichotomy effects to the effects of simply increasing S by the number of L cells. It is of some interest to have that "intuition" (a) tested (it may very well be flawed) and (b) made more precise with numerical estimates and specific behavioral characteristics. The value of the latter to a reader depends on how convincing they find the arguments, particularly with respect to alternative models, so I think the authors should go as far as possible in putting forward those arguments.

Overall, I believe that further clarification of the connection between the model and what is known experimentally, together with further exploration of assumptions and merits of the model, would add value to a publication. For a reader with my background, I predict that the value of this work would be greatly increased by the addition of an experimental test of the virtues of the model. Since such tests are not even discussed and may not be easy to specify, I am not considering them as likely revisions.
Smaller notes:
The title should be something like "Mathematical modeling suggests that licensing and competition….". This is clear in the Abstract but the title suggests that the suggestion has been proven.
"Self-renewal" is ambiguous. It may be appropriate to say "cell division" in some cases and to talk about lineage maintenance ion others.

© 2025. Published by The Company of Biologists under the terms of the Creative Commons Attribution License (https://creativecommons.org/licenses/by/4.0/).

Similarly, “division” is generally more literal than “proliferation”.
P16 Reference [30] is quoted to say there are 14 naiive cells based on clonal labeling. I see 13 in that paper. It is stated that contact tested with surface GFP led to an estimate of 18 cells contacting the hub. I read 13 in the paper.

First revision

Author response to reviewers' comments

The work presents a model for differentiation of cyst stem cells in *Drosophila* spermatogenesis. This model includes the phenomenon of ``licensing", for which experimental evidence has been provided in the past. Licensing means that stem cells become primed for differentiation, attaining a state ”licensed” for differentiation, from which they may commit to terminal differentiation, but they also retain the potential to return to the (naive) stem cell state, also called de-licensing. One particular additional constraint is that cyst stem cells can only commit to differentiation in pairs, by simultaneously engulfing a germline stem cells in a cyst. The authors study a stochastic version of the model, backed up by deterministic equations for mean values in the Supplemental Information. They notice a certain degree of robustness to perturbations in the model, which is interpreted as a stable homeostatic state, yet stochastic fluctuations can lead to extinction of the stem cell population in some cases. The authors claim that the stability of homeostasis is the result of (de-)licensing. Through addition of competition for niche space, realistic rates of extinction and fluctuation variances, that match experimental data, can be achieved. The manuscript is well written and the modelling results seem robust and correct. The model is well motivated by experimental observations and is likely capturing the relevant features of the real world scenario. When spatial competition is taken into account, its predictions can be brought in agreement with experimental data, which further confirms the model assumptions. The importance of this work is that the model incorporates mechanisms to confer stable homeostasis in spermatogenesis (and possibly other systems). However, I am convinced that the authors misinterpreted the particular mechanism within the model which is responsible for maintaining homeostasis. Regarding this, see my further comments below."

Reply: We thank the reviewer for conducting a thorough review of our manuscript and helping us to improve it. We answer the questions point by point below.

"I do have doubts about the claim that homeostasis (as in, a stable steady state of expected cell numbers) is the result of (de-)licensing. I am rather convinced that the key ingredient to confer stable homeostasis is the fact that commitment to differentiation occurs through pairwise interaction between cells. In fact, (de-)licensing is not required per se for that. This can be seen when analysing the deterministic equation for the mean cell numbers, Eqs. (9,10) in the Supplemental Information. If differentiation of licensed cells would not occur pairwise, the last term of Eq. (10) were linear, and in fact the whole system were linear. In that case, no non-zero asymptotically stable state can exist, only possibly a neutrally stable state which features diverging fluctuations over time. In addition to diverging fluctuations, such a neutrally stable state could only be achieved through accurate fine-tuning of proliferation vs. differentiation rates. Thus, the pairwise interaction is necessary for stable homeostasis. The authors claim that without (de-)licensing homeostasis does not prevail. However, this is only because without (de-)licensing, commitment to terminal differentiation, in that case the transition from stem cells to L-cells (which are not ”licensed” anymore in that scenario), occurs before the pairwise differentiation. If instead, in that scenario, commitment would be through pairwise interaction, that is, involving two naive stem cells, then we would have again a stable homeostatic state. This can be seen in Eq. (25) of the Supplemental Information, when we would have dn_S/dtau = n_s - alpha*n_s^2, which yields a stable steady state
for alpha >1.

Hence, the crucial mechanism to enable stable homeostasis is that, at the point were cells irreversibly commit to terminal differentiation, they do so via pairwise interaction (any other non-

© 2025. Published by The Company of Biologists under the terms of the Creative Commons Attribution License (https://creativecommons.org/licenses/by/4.0/).

linear interaction could work as well), irrespective of whether (de-)licensing occurs or not. (De-)Licensing affects this only in so far, as it moves the point of irreversible commitment further down the hierarchy. I therefore think that the claim that stable homeostasis is caused by (de-)licensing cannot be upheld, and that the authors should adapt their main claim and to consider more precisely the effect of the pairwise interaction on homeostatic control. This would likely also require to change the title, which includes the claim. The competition for finite niche space, on the other hand, is a further viable mechanism to maintain a stable homeostatic state, and to reduce fluctuations in cell numbers."

Reply: We thank the reviewer for raising this interesting point. Indeed, the stability of the homeostatic state in the SL and vSL models depends of a combination of pairwise cyst formation (followed by irreversible differentiation) and the presence of licensing/de-licensing rendering the cyst formation the sole irreversible step in our system. Our statement that licensing alone is sufficient regulation to maintain homeostasis takes the presence of pairwise differentiation events for given. In that scenario, the ability of stem cells to reversibly transition between naive and licensed states is crucial to maintain a stable steady state. We agree that in a scenario where naive stem cells can differentiate in pairs without the presence of licensed states, stable homeostasis would be also achieved. However, that scenario would require CySCs remaining as naive stem cells in the region where cysts are formed, which is highly unlikely as CySCs require niche signals to remain in such a state and these signals likely do not extend into the cyst formation region. Indeed, our model with naive and licensed states is fairly equivalent to a model with only naive stem cells that can differentiate in pairs, but with a larger naive stem cell pool size compared to what is biological plausible. It can be interpreted that the role of licensing is to increase the effective stem cell pool size. We have modified our manuscript to reflect that licensing is effective at regulation when it acts in combination with pairwise differentiation, and added paragraph in the Discussion section (paragraph 2) to reflect that.

"Beyond this concern, I have only one further suggestion: it would be good to see the results for the vSL model in a figure, analogue to Fig. 3 for the SL-model. Currently, the results of the vSL model are less clear
without visualisation

Reply: We have added a new figure (Figure 5 in the revised manuscript) showing stochastic simulations and the quasi-steady state distributions for different parameter values of the vSL model.

Reviewer 2
" A mathematical model is developed for a community of stem cells with two different states, named naiive and primed, which can exchange. Additionally, only primed cells can directly differentiate. The model is mostly potentially general but is being applied to the Drosophila testis and therefore has a specialized component that differentiation of primed cells only occurs in pairs (which must be spatially close and in contact with a germline gonialblast, also ready to differentiate). The effect of dividing stem cells into two states was compared to a single state in the context of no other constraints or when an additional feature, competition for niche access, is introduced by modeling using some experimental measurements of average populations of the two stem cell states. The reported results are that the extra state reduces modeled fluctuations in stem cell numbers (but not sufficiently to reflect experimental observations) and supplements the effect of niche competition alone, so that extinctions of the stem cell population are less frequent and compatible with observations."

Reply: We appreciate the insightful comments from the reviewer, which have significantly contributed to enhancing our manuscript. We answer the questions point by point below.

"This reviewer has paid some attention to modeling but does not have expertise to evaluate the modeling details per se or to compare in a detailed way to other relevant modeling studies. My perception is that the use of mathematical models might have three major components- (i) formulation of the model according to sufficient (never complete) experimental knowledge of key parameters, (ii) assumptions (mostly simplifying to assume certain factors are constant, that others may not be limiting, or that there are discrete states rather than a continuum or multiplicity of states) that allow creation of a relatively simple model to examine predicted or simulated cell

© 2025. Published by The Company of Biologists under the terms of the Creative Commons Attribution License (https://creativecommons.org/licenses/by/4.0/).

behavior, and ideally (iii) making an alteration to the experimental system that tests a prediction of the model or shows its benefit.

I have some significant reservations in all three areas. Most straightforward, there is no testing of the model by making one or more specific experimental perturbations. For me, this is a serious deficiency. No model is going to be truly accurate, but why should anyone take special note of this model if it has not proven its value in this way. The modeled system affords many genetic tests. If it is possible to test the model well, surely this is the place to report that.

Reply: We thank the reviewer for their suggestion. We have conducted an additional experiment to test the vSL model predictions for the recovery dynamics against experimental data. Our experiment consists of starving Drosophila for 14 days, leading to the loss of a fraction of CySCs, followed by re-feeding, and measuring the number of CySCs in the subsequent days. We report our findings in a new Results section (F), and through a new figure (Fig. 7), and show overall agreement between the experiment and our model predictions.

"The connection between experimental observations and the formulation of the model needs some further clarification. The model appears to be framed according to which cells contact the niche. I believe that translates to primed cells being “two diameters” from the niche and not in contact. This should be confirmed. A potentially important point here is whether this simple duality appears to exist in reality. Is there not evidence for different degrees of contact and for different expression of reporters of signals deriving from the hub niche (and a ”differentiation niche”) for a given nuclear location (1 vs 2 cell diameters away)?

Reply: The reviewer is correct, we equate licensed cells with those located two cell diameters from the hub and therefore not in contact with the hub. There is substantial evidence for dual states and not graded contact. Firstly electron micrographs show that only a subset of somatic cells contact the hub, these are those present in the first row [4]. Clones in which the cells were labelled with a membrane-targeted GFP show the same result, with only &[approx]30\% of Zfh1-expressing cells contacting the hub [1]. Activity of the JAK/STAT pathway, the main self-renewal pathway in CySCs is only observed in the first row ([5] and many others), while activity of Tor is detected mainly in the second row [2], although occasionally in the first row too (in the 2016 paper, we report an average of 1.6 Tor-positive cells in the first row). Notably, apart from the differential signalling, there are very few transcriptional differences between the two rows, and most markers used label both populations, supporting the idea that these are different states of the same cell type, determined by the level of signalling they experience.

" The model includes cells 2 diameters away sometimes moving back to contact the hub. The model seems mainly built on the basisof reference [5], wherein Amoyel et al, 2014 is used to support the statement that “CySC replacement by neighbors is a frequent event”, then noting that CySCs are identified by contact with the niche. This is not equivalent to evidence of cells moving from “layer 2” to “layer 1” and there are no such data in the 2014 paper.
"

Reply: The reviewer is correct, reference [5] of the manuscript does not show directly that CySCs move from the second to the first row. However, there is evidence in the literature to supoprt this claim [3]. We have conducted an additional experiment to further illustrate that CySCs often migrate from the second to the first row. This experiment aims to trace the lineage of licensed cells by performing Spict lineage and using the fact the regions of high Tor activity and Spict expression are correlated. We induce GFP in licensed cells and measure the presence of the label in the first row at subsequent times. We observe an increasing number of GFP-positive cells in the first row after induction, consistent with frequent de-licensing events. This results are in-line with previous studies [3]. We added a new section in the SI reporting the results of this experiment.

"While there is prior evidence that JAK-STAT and some other signals are higher in cells next to the niche, while the PI3K pathway is higher in more distant cells, and that the former sets of signals affect division rate, while the latter contributes to final differentiation, there seem to be numerous uncertainties also (as would be expected). For example, do any of these signals also affect the movement of cells from one layer to another? In other words, are the circumstances (signals, geometry) often affecting more than one model parameter? If so, can each parameter really be

© 2025. Published by The Company of Biologists under the terms of the Creative Commons Attribution License (https://creativecommons.org/licenses/by/4.0/).

considered independent? It seems particularly difficult to exclude geometry. First, the potential for final differentiation depends on the availability of a neighboring “licensed” cell and a suitable germline cell. Both of these emerged locally from stem cells that were competing for niche access. Second, the likelihood of a cell returning to contract the hub depends on available space. This will depend on the state of various nearby cells (division of cells at the niche, attempted return of neighbors). Third, transition of S L could similarly depend on the density of niche-adjacent cells.

In the end, a model introduces competition for the niche as an additional factor but my understanding is that this does not negate these concerns about independent parameters of the basic “SL” model."

Reply: This study analyses what parameter regimes yield a homeostaic behaviour that is compatible with experimental observations (size of homeostatic fluctuations during homeostais and recovery dynamics), but remains agnostic to the plausible independence of our parameters. The question of whether there are master regulators affecting one or more parameter at the same time is an interesting avenue for future research. The results and analysis of this study, however, will remain valid in any case.

Note that our model does include co-dependencies between parameters due to, for example, geometrical factors. For example, a reduction of the carrying capacity in the vSL model also affects the effective proliferation and cyst formation rates. We agree with the reviewer's observations of the dependencies of cyst formation and licensing rates on the available space in the niche, and these dependencies are reflected in our model. For example, in our vSL model an increase of available space in the niche implies a reduction of the number of naive stem cells, which decreases licensing and increases the de-licensing rate, leading to fewer licensed CySC and a lower rate of cyst formation.

"Regarding the model itself,
1. I find it is laid out understandably for the non-expert. I am interested in the authors’ opinion on some other models (tangential to the main topic), which consider the frequency of outcomes of cell divisions (producing two stem cells, two non-stem cells or one of each) even when the paradigm does or may actually involve separate division and differentiation events. I have always thought that is inherently flawed and am glad to see the formulation presented here is different.

Reply: For the sake of mathematical tractability, various models consider that the fate of stem cells is decided upon division. While this may be true for certain tissues, the assumption breaks down in cases where differentiation requires active signalling or other environmental cues, which might be the case for the majority of tissues. A model in which stem cells can either divide symmetrically, into two stem or differentiated cells, or asymmetrically, imposes a correlation between proliferation and differentiation events that might not be present, with special implications in the behaviour of the system outside homeostasis. Other studies argue that for a mutation that increases the proliferation rate to out-compete other clones in a stem cell niche, division and differentiation must be de-coupled [6].

"2. The assumption that germline cell status is not relevant seems fragile- germline status might be instructive and limiting. The omission of spatial relationship between two potentially differentiating cells, I think, effectively assumes that an average separation will be uniform, which seems very unlikely."

Reply: The reviewer makes an important point and indeed, we agree that the availability of germ cells could be a restriction on CySC differentiation. However, we provide evidence from time lapse movies (Supplemental Movie S1) that licensed CySCs are highly motile and move around the niche laterally, contacting several germ cells as they do so. Thus, it appears that CySCs may not be limited by the need for a germ cell immediately adjacent to them. We agree that the question of how the availability of germline stem cells affects the cyst formation and, consequently, the dynamics of the CySC population is interesting, and will be an object of future research. We regard this study as a necessary previous step for the analysis of a more complex model that includes both the CySC and germline populations.

© 2025. Published by The Company of Biologists under the terms of the Creative Commons Attribution License (https://creativecommons.org/licenses/by/4.0/).

"3. It seems that there could just as easily be several states, rather than just S and L. I understand that simplification and seeing what just two states can accomplish has merits, but this leaves open the question of what might be added by multiple states."

Reply: This is an interesting question for future research. Our choice of two CySCs states stems from the evidence of the existence of two clearly identifiable populations, one that can proliferate, is in contact with the hub and has JAK/STAT activity, and one that does not proliferate, can form cysts and has high PI3K/Tor activity (see also reply # 3 to Reviewer 2). We agree that a possible refinement of the model may consider the existence of intermediate states between naive and licensed, or even a population with continuous distributions of the propensities for proliferation and cyst formation. However, we do not have evidence suggesting how these internal states might be distributed or what behaviours would characterise them, and such a refinement would render the models mathematically intractable. The propensities and rates for proliferation, licensing, de-licensing and cyst formation in our model represent averages across a likely more heterogeneous population.

"4. The chosen method for adding niche competition is presumably not the only modeling choice, nor is it necessarily true that niche competition is really a single process (for example, since signal strengths may play complicated roles, competition for a certain space with niche contact may not be the only relevant, operative parameter). I am therefore suspicious of whether the S/L division is just one of several possible arrangements (compatible with reality) that complement and add to properties endowed by niche competition. Specifically, the niche competition method talks about empty space or not, as black or white, rather than degrees of space available. It would help to clarify whether introducing a variable space is likely in reality or practical in a model. "
and
" 5. I think it would be very useful to compare the authors' model with various other published models dealing with the same issues of stabilizing stem cell populations. It seems to me there is considerable overlap- hence, it is appropriate to have expert comment.
"

Reply: We thank the reviewer for raising this interesting point. We agree that competition for niche access is not the only possible mechanism related to the niche that might be active. For example, competition for other resources, such as mitogens or fate determinants, might occur at the same time. During homeostasis, competition for niche access and for mitogens may be indistinguishable. Perturbation experiments could help distinguishing between both mechanisms. Identifying a scenario where both competition for niche access and for mitogens are present may be challenging even from perturbation experiments, and may require blocking one of the mechanisms to show the other is still present. We have added an additional paragraph (paragraph 4) in the Discussion briefly comparing our models with alternative models in the literature.

"It is a detraction that the model is tailored to one paradigm. How would the model differ if applied, for example, to the mouse small intestinal crypt?"

Reply: We briefly discussed this in the last paragraph of the Discussion. We have added a new paragraph in the Discussion (paragraph 7) discussing modifications of the model for the intestinal crypt.

"Finally, I would say that it seems intuitively obvious that having an intermediate state with reversible exchanges will improve reliability. One way is simply by increasing the total population of stem cells. It may be useful to compare the S/L dichotomy effects to the effects of simply increasing S by the number of L cells. It is of some interest to have that "intuition" (a) tested (it may very well be flawed) and (b) made more precise with numerical estimates and specific behavioral characteristics. The value of the latter to a reader depends on how convincing they find the arguments, particularly with respect to alternative models, so I think the authors should go as far as possible in putting forward those arguments."

Reply: The reviewer raises an interesting point. In a system comprised solely of naive stem cells capable of forming cysts, the homeostatic population of these naive stem cells required to attain a cyst formation output comparable to that observed in the SL and vSL models exceeds the

© 2025. Published by The Company of Biologists under the terms of the Creative Commons Attribution License (https://creativecommons.org/licenses/by/4.0/).

homeostatic number of naive CySCs of the latter, but is lower than the total number of CySCs. We have added a paragraph in the Discussion (paragraph 2) commenting on this.

"Overall, I believe that further clarification of the connection between the model and what is known experimentally, together with further exploration of assumptions and merits of the model, would add value to a publication. For a reader with my background, I predict that the value of this work would be greatly increased by the addition of an experimental test of the virtues of the model. Since such tests are not even discussed and may not be easy to specify, I am not considering them as likely revisions.

Smaller notes:
The title should be something like 'Mathematical modeling suggests that licensing and competition….'. This is clear in the Abstract but the title suggests that the suggestion has been proven. 'Self-renewal' is ambiguous. It may be appropriate to say “cell division” in some cases and to talk about lineage maintenance ion others. Similarly, “division” is generally more literal than 'proliferation'.
"

Reply: We modified the title and changed the nomenclature.
" P16 Reference [30] is quoted to say there are 14 naiive cells based on clonal labeling. I see 13 in that paper. It is stated that contact tested with surface GFP led to an estimate of 18 cells contacting the hub. I read 13 in the paper."

Reply: The average of the naive cells based on the clonal labelling experiments is 13.5 with a standard error of 0.7. In parallel, the estimates using a membrane-targeted GFP yields an average of 18.3 with standard error of 0.2. Therefore, we considered that the homeostatic number of naive CySCs falls within 13 and 19, and we assumed uncertainty within this range. Our results and conclusions remain valid for all values within this range. We discussed this point in the Methods section.

References

[1] Marc Amoyel, Benjamin D Simons, and Erika A Bach. “Neutral competition of stem cells is skewed by proliferative changes downstream of Hh and Hpo”. In: The EMBO journal 33.20 (2014), pp. 2295–2313.

[2] Marc Amoyel et al. “Somatic stem cell differentiation is regulated by PI3K/Tor signaling in response to local cues”. In: Development 143.21 (2016), pp. 3914–3925.
[
3] Ason C-Y Chiang, Heiko Yang, and Yukiko M Yamashita. “spict, a cyst cell-specific gene, regulates starvation-induced spermatogonial cell death in the Drosophila testis”. In: Scientific reports 7.1 (2017), p. 40245.

[4] RW Hardy et al. “The germinal proliferation center in the testis of Drosophila melanogaster”. In: Journal of ultrastructure research 69.2 (1979), pp. 180–190.

[5] Melanie Issigonis et al. “JAK-STAT signal inhibition regulates competition in the Drosophila testis stem cell niche”. In: Science 326.5949 (2009), pp. 153–156.

[6] Amy Reilein et al. “Division-independent differentiation mandates proliferative competition among stem cells”. In: Proceedings of the National Academy of Sciences 115.14 (2018), E3182–E3191.

© 2025. Published by The Company of Biologists under the terms of the Creative Commons Attribution License (https://creativecommons.org/licenses/by/4.0/).

Second decision letter

MS ID#: DEVELOP/2024/202796

MS TITLE: Licensing and Niche Competition in Spermatogenesis: Mathematical Models Suggest Complementary Regulation of Tissue Maintenance

AUTHORS: Rodrigo Garcia-Tejera, Jing-Yi Tian, Marc Amoyel, Ramon Grima, and Linus Schumacher

I have now received all the referees reports on the above manuscript, and have reached a decision. The referees' comments are appended below, or you can access them online: please go to BenchPress and click on the 'Manuscripts with Decisions' queue in the Author Area.

The overall evaluation is positive and we would like to publish a revised manuscript in Development, provided that the referees' comments can be satisfactorily addressed. Please attend to all of the reviewers' comments in your revised manuscript and detail them in your point-by-point response. If you do not agree with any of their criticisms or suggestions explain clearly why this is so. If it would be helpful, you are welcome to contact us to discuss your revision in greater detail. Please send us a point-by-point response indicating your plans for addressing the referees' comments, and we will look over this and provide further guidance.

Reviewer 1

Advance summary and potential significance to field

The authors addressed my concerns reasonably. In the abstract and discussion, they now correctly point out the crucial role which the pairwise interaction at irreversible differentiation plays in maintaining homeostasis.

It is correct that licensing is possibly important to effectively extend the stem cell pool beyond the immediate niche, towards cellular environments where cyst development occurs. In that aspect, licensing confers additional *robustness* to the system, in that structural disruptions of the system are easier to be compensated. However, I still find that the role of licensing in conferring *stability* per se, that is towards perturbations of cell numbers, is overstated. As the authors correctly note in their reply, licensing is, from a mathematical standpoint, not necessary nor sufficient to ensure stability, while pairwise interaction is both necessary (in the context of this model; other models could of course provide other stability mechanisms) and sufficient, if it occurs at the point of irreversibility. I do agree that biological constraints other than the maintenance of homeostasis alone, may render licensing necessary, but this should not be mixed with the quantitative mechanism to confer stability.

Comments for the author

I would recommend to clarify a bit more the different roles of licensing and pairwise differentiation, the former improving robustness towards structural disruptions and an effective biological expansion of the stem cell pool, the latter providing 'classical' stability towards number perturbations.

Reviewer 2

Advance summary and potential significance to field

Omitted since this is a re-review.

© 2025. Published by The Company of Biologists under the terms of the Creative Commons Attribution License (https://creativecommons.org/licenses/by/4.0/).

Comments for the author

The comments are below. I want to draw attention to the Summary section, with respect to one of the mandatory reviewer questions- I would like to see a response but, if the other reviewer's concerns have been met, I do not think it is necessary for me to evaluate the response before the editor accepts the manuscript.

It is quite difficult to organize my comments in a simple logical thread with the most important first, so I will recapitulate my thought processes.

1. The current task is to address the adequacy of revisions, having already ascribed sufficient value to encourage a revision. I found the general tone of responses to be thoughtful and productive. I am interested, as a by-stander, in the adjustments to the qualities imparted to a model by each named parameter, but I will leave judgment to experts in the modeling and mathematics. The authors added a recovery-from-starvation experiment and modeling. I had not expected this type of addition (despite my request for testing predictions experimentally). Indeed, I worry about last-minute additions in case they are not as carefully considered as longer-standing results, so I hope the authors are confident about the addition. I do not see that the concordance with experiment shows a unique attribute of the model (i.e., other models fail) and the protocol involves such dramatic changes that all manner of changes in cell states versus position over time may be possible. In other words, the effort is appreciated but I would not insist on retaining this addition to achieve requisite value for publication. Numerous initial review comments were to provoke clarifications or additional explanations. These were mostly provided in good faith. There, nevertheless, remain, a few issues of clarity and wording (old or new) that could beneficially be addressed. One particular response and addition provoked an important set of thoughts (“2” below).

2. The initial question was about the evidence for saying that “licensed stem cells” frequently return to make hub contact.

(a) Evidence
The response cited a paper using spict-GAL4 and partial repeat of the same lineage test.
The GFP staining in the repeat images seems far from ideal and makes me wonder how quantitation was possible. The result was similar to the prior paper, albeit with slightly different timescale of recovery. I am on the fence about the merits of this addition, given the GFP staining.
The bigger issue for me is that I think the experimental approach is flawed. The idea of the experiment is to have a pulse-chase: label only “licensed cells”, prevent further labeling, see what happens to the labeled cells over time. In fact, labeling is initiated by switching to 29C and chased at 29C to allow continuous labeling. The flaw would also be present if flies were quickly returned to 18C, if the 29C pulse were short and if only a small fraction of “licensed cells” were labeled in each sample. Time zero would also have to allow an interval sufficient for recombination to take place, GFP to build up and be detectable. Unfortunately, these events will not be synchronized among cells, so a perfect approach is not possible. However, with the chosen approach there is a significant chance that some labeling events occur in cells touching the hub because there is a limit to the conceivable difference in spict-GAL4 expression between the two cell populations. If conditions allowed for only a few cells to be labeled this would much better bias labeling towards stronger spict-GAL4 expression.
The question of how frequently (roughly) cells regain hub contact seems to me very important for the field. So, I think it is worth doing a better experiment. If not, I think it is appropriate to describe reservations (fairly, not grudgingly).
Either way, can the current experiments or a better experiment provide a value (range) for the average frequency of return? I think this calculation or its limitations should be explained.

(b) Implications for names
Looking at Chiang et al (I regret not having seen this at the time of the first review), I see that the return of “licensed cells” to the hub was presented as reconciling (i) observation that CySC divisions immediately produced one daughter contacting the hub (Cheng et al, 2011) and (ii) population asymmetry lineage dynamics for MARCM lineages (Amoyel, Simons, Bach).

© 2025. Published by The Company of Biologists under the terms of the Creative Commons Attribution License (https://creativecommons.org/licenses/by/4.0/).

Curiously, I see that the latter paper states “there is no evidence for oriented division among CySCs (Cheng et al., 2011)…” This seems like a major error deserving an Erratum (EMBO J), which one of the authors of this submission could request.
I expressed reservations about the key “evidence of return”. I will admit that the result find does seem to reconcile the two observations. I would also note that in MARCM, even though a dividing cell undergoes recombination, the marked derivative could be either of the daughters. Hence, population asymmetry behavior is reporting the sum of the two locations of marked cells. If “licensed cells” indeed frequently return to hub contact it is clear that both locations of marked cells serve as stem cells. This is consistent with the current manuscript, naming naiive and licensed stem cells but it is at odds with some other statements and many prior publications, which have taken hub contact as defining a CySC and treated those cells as the only stem cells. I think it would be advantageous to describe these key results and the naming of stem cells. I believe it is highly likely that most readers or investigators consider only the hub-contacting cells as stem cells and that it is valuable to spell out the contrary evidence (assuming the spictGAL4 results are, to a degree, valid).

(c) How does any of the above impact investigations or conclusions in this paper?
The simplest question I have is to understand how (numerically) the frequency of return impacts the model. I understand that it is a key aspect of the model and cannot therefore be negligible but it would be good to understand what range of values has an impact (presumably comparing to average rate of CySC division).
Related to this, the authors responded to my question about the properties of a model where there is no division among stem cells (all 30-40 Zfh-1 cells are equivalent stem cells) [essentially, division is not restricted to one subset]. The answer (in response and in the Discussion) is ambiguous – “the stem cell pool size would have [to] be larger” and “beyond what is biologically plausible”. Please state how large. I assume that the “return rate” would affect this same issue.

3. A collection of small points Abstract: “regaining stem cell function” (repeated in Intro) Isn’t this resuming cell division & would it not be crucial to describe this in terms of location? The “licensed cells” are part of the stem cell population, as the authors acknowledge with their names.
Before “We find that licensing…” I think a reader may need to know that the potential problem of stochastic systems is variability in stem cell numbers or extinction (which the authors take for granted).
“is sufficient” This is always problematic. What does it actually mean here? Later, Zfh-1 is quoted as sufficient for stem cell behavior. Since most authors only consider CySCs as stem cells (likely mistakenly), this is clearly a mis-statement of general beliefs.
“Experimental data further suggests”- sounds like a new experiment- “have shown?” (unless there is remaining doubt), then “we show….” Should surely include something about theory or modeling.

P5 “we are assuming abundance of germline stem cells” Aren’t gonialblasts the relevant cells? The movie is nice (label different “unmarked” germline cells you claim are contacted at different times?) but I think this hardly dismisses the issue.

The model talks about rates. Please include whatever estimate is available about CySC division rate, cyst production rate and (from earlier) rate of return.

In the new recovery from starvation experiment the total number of CySCs is 32.4 & said to be as usually reported. I am not clear why this differs from the number of Zfh-1 positive cells and does not agree with the idea of about 16 hub-contacting cells representing so% of the stem cell population.

Discussion of other paradigms:
Intestinal stem cell- the potential parallels are perhaps murky in light of recent evidence for (some of) the population previously considered TAs to be part of the stem cell pool. This should be referenced. It is arguable to what degree it is relevant to the current models.

I think that another interesting paradigm deserves mention.
In essence, the key modeling features for heterogeneity in the testis among stem cells involve cell locations which differ by (a) division and (b) immediate conversion to a differentiated cell. The same type of organization (but opposite division rate polarity) exists for Drosophila ovarian FSCs.

© 2025. Published by The Company of Biologists under the terms of the Creative Commons Attribution License (https://creativecommons.org/licenses/by/4.0/).

The situation there is well-documented experimentally but the question of how or if it has specific benefits has not, I believe, been addressed.

Summary:
I hope that the authors will make appropriate revisions in response to these comments. From my reviewer perspective, I trust those will be made and would not require that I find those responses satisfactory for acceptance. I would nevertheless be interested to see what the responses are, continuing what I believe has been a productive dialog for all.

Second revision

Author response to reviewers' comments

Reviewer 1
The authors addressed my concerns reasonably. In the abstract and discussion, they now correctly point out the crucial role which the pairwise interaction at irreversible differentiation plays in maintaining homeostasis.

It is correct that licensing is possibly important to effectively extend the stem cell pool beyond the immediate niche, towards cellular environments where cyst development occurs. In that aspect, licensing confers additional *robustness* to the system, in that structural disruptions of the system are easier to be compensated. However, I still find that the role of licensing in conferring *stability* per se, that is towards perturbations of cell numbers, is overstated. As the authors correctly note in their reply, licensing is, from a mathematical standpoint, not necessary nor sufficient to ensure stability, while pairwise interaction is both necessary (in the context of this model; other models could of course provide other stability mechanisms) and sufficient, if it occurs at the point of irreversibility. I do agree that biological constraints, other than the maintenance of homeostasis alone, may render licensing necessary, but this should not be mixed with the quantitative mechanism to confer stability.

I would recommend to clarify a bit more the different roles of licensing and pairwise differentiation, the former improving robustness towards structural disruptions and an effective biological expansion of the stem cell pool, the latter providing 'classical' stability towards number perturbations.

Reply: We thank the reviewer for pointing out that the contribution of licensing and the fact that differentiation takes place in pairs to the stability and robustness of the system could be clarified further. We agree that the key mechanism providing the system with stability is the pairwise differentiation, with de-licensing contributing to the extension of such stability to the naive stem cell pool by eliminating the irreversibility of licensing events. We modified the manuscript to further clarify the role of licensing in providing robustness to the system, while emphasising that the key mechanism conferring stability is the pairwise differentiation. Specifically, we modified the subsection headings of the Results section to emphasise that the mechanism of interest is the combination of pairwise differentiation and licensing/de-licensing, and we extended the last paragraph of subsection A of the Results section.

Reviewer 2
The comments are below. I want to draw attention to the Summary section, with respect to one of the mandatory reviewer questions- I would like to see a response but, if the other reviewer's concerns have been met, I do not think it is necessary for me to evaluate the response before the editor accepts the manuscript.

It is quite difficult to organize my comments in a simple logical thread with the most important first, so I will recapitulate my thought processes.

1. The current task is to address the adequacy of revisions, having already ascribed sufficient value to encourage a revision. I found the general tone of responses to be thoughtful and productive. I

© 2025. Published by The Company of Biologists under the terms of the Creative Commons Attribution License (https://creativecommons.org/licenses/by/4.0/).

am interested, as a by-stander, in the adjustments to the qualities imparted to a model by each named parameter, but I will leave judgment to experts in the modeling and mathematics. The authors added a recovery-from-starvation experiment and modeling. I had not expected this type of addition (despite my request for testing predictions experimentally). Indeed, I worry about last-minute additions in case they are not as carefully considered as longer-standing results, so I hope the authors are confident about the addition. I do not see that the concordance with experiment shows a unique attribute of the model (i.e., other models fail) and the protocol involves such dramatic changes that all manner of changes in cell states versus position over time may be possible. In other words, the effort is appreciated but I would not insist on retaining this addition to achieve requisite value for publication.

Reply: The reviewer is correct that the starvation and re-feeding experiment alone does not necessarily exclude alternative regulatory mechanisms, as these would have to compared separately and formally to the data and our current model's prediction. However, the primary aim of this experiment is to test the predictive ability of our vSL model, which was developed based on observations of the stem cell behaviour in homeostasis, outside steady-state conditions. We believe that the alignment between the experimental results and the model's predictions enhances the reliability of our model.

Numerous initial review comments were to provoke clarifications or additional explanations. These were mostly provided in good faith. There, nevertheless, remain, a few issues of clarity and wording (old or new) that could beneficially be addressed. One particular response and addition provoked an important set of thoughts ("2" below).

2. The initial question was about the evidence for saying that "licensed stem cells" frequently return to make hub contact.

(a) Evidence
The response cited a paper using spict-GAL4 and partial repeat of the same lineage test.
The GFP staining in the repeat images seems far from ideal and makes me wonder how quantitation was possible. The result was similar to the prior paper, albeit with slightly different timescale of recovery. I am on the fence about the merits of this addition, given the GFP staining.

Reply: We agree that GFP levels do vary between cells (and are consistently lower in the first row - we suspect this is related to specific translational control in CySCs in response to signals from the niche as we have observed this with other GFP reporters expressed uniformly in the cyst lineage). However, given the nature of the experiment, in which DNA is permanently altered through recombination for GFP to be expressed, levels can be ignored, and cells can be accurately scored as GFP-expressing or non-expressing. Overall, the point of adding this quantification was to show that exchange of cells between the first and second rows does occur frequently.

The bigger issue for me is that I think the experimental approach is flawed. The idea of the experiment is to have a pulse-chase: label only "licensed cells", prevent further labeling, see what happens to the labeled cells over time. In fact, labeling is initiated by switching to 29C and chased at 29C to allow continuous labeling. The flaw would also be present if flies were quickly returned to 18C, if the 29C pulse were short and if only a small fraction of "licensed cells" were labeled in each sample. Time zero would also have to allow an interval sufficient for recombination to take place, GFP to build up and be detectable. Unfortunately, these events will not be synchronized among cells, so a perfect approach is not possible. However, with the chosen approach there is a significant chance that some labeling events occur in cells touching the hub because there is a limit to the conceivable difference in spict-GAL4 expression between the two cell populations. If conditions allowed for only a few cells to be labeled this would much better bias labeling towards stronger spict-GAL4 expression.
The question of how frequently (roughly) cells regain hub contact seems to me very important for the field. So, I think it is worth doing a better experiment. If not, I think it is appropriate to describe reservations (fairly, not grudgingly).
Either way, can the current experiments or a better experiment provide a value (range) for the average frequency of return? I think this calculation or its limitations should be explained.

© 2025. Published by The Company of Biologists under the terms of the Creative Commons Attribution License (https://creativecommons.org/licenses/by/4.0/).

Reply: The reviewer raises an excellent point. It is indeed possible that a small fraction of CySCs contacting the hub do undergo recombination induced by Spict-Gal4 driven Flippase. This could be either leakage of the system or due to occasional real expression of Spict-Gal4 in first row cells. Nonetheless, the experiment, together with other data, supports the idea that cells in the second row do contribute to the first row at a non-negligible rate. For instance, while many labs have reported that mitosis only occurs in CySCs in contact with the hub (eg 10.1242/dev.057901), DNA replication is seen in cells in both the first and second rows (see for example 10.1016/j.celrep.2022.110774), implying that cells that replicate their DNA in the second row return to the first row to undergo mitosis.
Regarding calculating the frequency of return, this would indeed be nice; however, this is not trivial, due to the fact that returned cells (which are permanently labelled through DNA recombination, meaning all their offspring are labelled too) can divide. Along with allowing for the possibility of leakage, this makes calculating a rate extremely challenging. Finally, it is worth noting that ongoing work in the Amoyel lab, led by Jing-Yi Tian who did the experimental work in this manuscript, is currently trying to estimate such parameters from live imaging. Given the challenging nature of that work, this would form part of a separate manuscript.
As we indeed agree that the point raised is important, we have edited the manuscript text to point out the limitation of the lineage-tracing experiment (see Section SIV in the SI).

(b) Implications for names
Looking at Chiang et al (I regret not having seen this at the time of the first review), I see that the return of "licensed cells" to the hub was presented as reconciling (i) observation that CySC divisions immediately produced one daughter contacting the hub (Cheng et al, 2011) and (ii) population asymmetry lineage dynamics for MARCM lineages (Amoyel, Simons, Bach).
Curiously, I see that the latter paper states "there is no evidence for oriented division among CySCs (Cheng et al., 2011)…" This seems like a major error deserving an Erratum (EMBO J), which one of the authors of this submission could request.

Reply: Whether CySCs do divide asymmetrically or not has been the subject of debate in the field for some time. However, the statement in the 2014 EMBO J paper is correct: Cheng et al showed that the spindle of CySCs is not oriented relative to the hub (unlike GSCs). They observed only that one of the two daughters inherited the attachment to the hub. However, their experiments did not assess the long-term implications on fate, while the Amoyel, Simons and Bach paper showed that CySCs produce symmetric outcomes with extremely high rates (1 symmetric outcome/stem cell/day). Thus, inheriting the attachment may not be relevant to determine fate.

I expressed reservations about the key "evidence of return". I will admit that the result find does seem to reconcile the two observations. I would also note that in MARCM, even though a dividing cell undergoes recombination, the marked derivative could be either of the daughters. Hence, population asymmetry behavior is reporting the sum of the two locations of marked cells. If "licensed cells" indeed frequently return to hub contact it is clear that both locations of marked cells serve as stem cells. This is consistent with the current manuscript, naming naiive and licensed stem cells but it is at odds with some other statements and many prior publications, which have taken hub contact as defining a CySC and treated those cells as the only stem cells. I think it would be advantageous to describe these key results and the naming of stem cells. I believe it is highly likely that most readers or investigators consider only the hub-contacting cells as stem cells and that it is valuable to spell out the contrary evidence (assuming the spictGAL4 results are, to a degree, valid).

Reply: Indeed, we agree that the Chiang et al paper manages to reconcile the short-term (asymmetric inheritance of CySC-hub attachment) and long-term (stochastic fates for the two daughters of a stem cell division) observations. The lineage trace would suggest that many CySC daughters do indeed lose hub attachment but that this does not determine their fate, as indicated by their ability to return to contacting the hub. We also agree that these results (and the model we present here) would result in changing what we define to be stem cells, in that cells in both the first and second rows retain stem cell potential. This is indeed one of the findings of this modelling, which concludes that the existence of licensed second row cells essentially results in a larger effective stem cell pool. We note that this idea is similar to that described in mammalian intestine where "retrograde flow" of cells can contribute to increasing the effective stem cell pool (10.1038/s41586-022-04962-0) or the mouse testis where stem cells can transition between two

© 2025. Published by The Company of Biologists under the terms of the Creative Commons Attribution License (https://creativecommons.org/licenses/by/4.0/).

states (10.1016/j.celrep.2021.109875). One of the main conclusions of this work is essentially to support a broader shift in how stem cells are defined, from being a unique cell at the top of a hierarchy to a looser collection of cell states that all together sustain regenerative potential.

(c) How does any of the above impact investigations or conclusions in this paper? The simplest question I have is to understand how (numerically) the frequency of return impacts the model. I understand that it is a key aspect of the model and cannot therefore be negligible but it would be good to understand what range of values has an impact (presumably comparing to average rate of CySC division).

Reply: If one changes the de-licensing rate while maintaining all other parameters constant, the equilibrium numbers of naive and licensed stem cells will vary, but the steady state remains stable (to perturbation). For example, for the model without competition for niche access, the SL model, the impact of the de-licensing rate on the homeostatic numbers is given by Equations (S6) and (S7) of the SI. These equations show that the size of the naive stem cell pool in homeostasis is directly proportional to the de-licensing rate, and the size of the licensed stem cell pool is proportional to the squared de-licensing rate. The analysis included in SI A reveals that the stability of the homeostatic state is not affected by the de-licensing rate. Analogous results are valid for the model with competition for niche access, the vSL model (see SI Sec. SIII).

Related to this, the authors responded to my question about the properties of a model where there is no division among stem cells (all 30-40 Zfh-1 cells are equivalent stem cells) [essentially, division is not restricted to one subset]. The answer (in response and in the Discussion) is ambiguous – "the stem cell pool size would have [to] be larger" and "beyond what is biologically plausible". Please state how large. I assume that the "return rate" would affect this same issue.

Reply: In a model with only naive stem cells, we can consider the stem cell pool size needed to achieve the same cyst production rate as the model with naive and licensed stem cells. In a model S -->2S; 2S --> 0, the cyst production rate is given by &[gamma] n^2, where &[gamma] represents the rate in which one pair of cells form a cyst, and n* is the homeostatic number of cells, i.e., the stem cell pool size. In the SL model, the cyst production rate is given by &[gamma] S -->2S; 2S --> 0 is the average number of licensed cells in homeostasis. If the rate of cyst formation per pair of cells remains the same in both scenarios, that is, if &[gamma] is the same in both cases, the stem cell pool in the system without licensed states would have to be as large as the number of licensed stem cells in the SL model (26 in the dataset analysed in the MS), thus larger than the naive stem cell pool size (18 in the dataset analysed in the MS). Note that this estimation does not take into account competition for niche access. Doing the same calculation for the vSL model would require making assumptions on how the carrying capacity of the system changes in the case where all stem cells are naive, potentially involving spatial models and parameter inference. We believe that the outcome of such a study goes beyond the scope of the current manuscript, and that it would not further support or challenge any of the conclusions of the article, and therefore decided to leave it out of this study.

3. A collection of small points
Abstract: "regaining stem cell function" (repeated in Intro) Isn't this resuming cell division & would it not be crucial to describe this in terms of location? The "licensed cells" are part of the stem cell population, as the authors acknowledge with their names.

Reply: We agree and have rephrased this as ``returning to the niche and resuming cell division" based on the reviewer's suggestion.

Before "We find that licensing…" I think a reader may need to know that the potential problem of stochastic systems is variability in stem cell numbers or extinction (which the authors take for granted

Reply: We are somewhat limited by the 180 word limit for the abstract, but have addressed this by adding the qualifier ``extinction from stochastic fluctuations".

© 2025. Published by The Company of Biologists under the terms of the Creative Commons Attribution License (https://creativecommons.org/licenses/by/4.0/).

“is sufficient” This is always problematic. What does it actually mean here? Later, Zfh-1 is quoted as sufficient for stem cell behavior. Since most authors only consider CySCs as stem cells (likely mistakenly), this is clearly a mis-statement of general beliefs

Reply: We respectfully disagree. In the abstract, sufficiency refers to the mathematical model, where it is well defined as the equations with the sufficient properties (here, licensing and differentiation in pairs) exhibiting the desired property (here, stability to stochastic fluctuations). Later on in the manuscript Zfh-1 is quoted as necessary and sufficient for self-renewal, with a reference to earlier published work, so the context is clear and we don't think this is problematic usage of the phrase "is sufficient".

“Experimental data further suggests”- sounds like a new experiment- “have shown?” (unless there is remaining doubt), then “we show….” Should surely include something about theory or modeling.

Reply: We agree and have rephrased this as: ``Experimental data have shown that stem cells are competing for niche access, which our mathematical models demonstrate contributes to the reduction of the variability of stem cell numbers but does not prevent extinction".

P5 “we are assuming abundance of germline stem cells” Aren’t gonialblasts the relevant cells? The movie is nice (label different “unmarked” germline cells you claim are contacted at different times?) but I think this hardly dismisses the issue.

Reply: We agree that the production rate of gonialblasts, rather than the number of GSCs themselves, may represent a limiting factor. However, because cyst cells are highly motile, we assume they can move around until they locate a gonialblast. We acknowledge that this is an approximation that may not hold under certain conditions. Developing a model that explores how gonialblast production rates affect cyst cell dynamics is indeed a compelling direction for future research. Addressing this issue would deserve a separate manuscript on its own, so we leave it for future work.

The model talks about rates. Please include whatever estimate is available about CySC division rate, cyst production rate and (from earlier) rate of return.

Reply: The reviewer raises and interesting question. For the proliferation rate, Amoyel et al. 2014 finds a proliferation rate of about 1 division per cell per day. We do not have an estimate for the licensing, de-licensing or cyst formation rates. However, our modeling enables expressing the values of such rates in terms of the average number of naive and licensed stem cells in homeostasis, and the niche carrying capacity. We have estimates for the average number of naive and licensed stem cells in homeostasis, as presented in the MS, but we can only derive a lower bound for the carrying capacity from our study. Future work may be able to estimate one of the rate constants through a combination of experimental data analysis and parameter inference, which would then lead to the estimation of all the other rates via the vSL model.

In the new recovery from starvation experiment the total number of CySCs is 32.4 & said to be as usually reported. I am not clear why this differs from the number of Zfh-1 positive cells and does not agree with the idea of about 16 hub-contacting cells representing so\% of the stem cell population

Reply: Different strains of Drosophila may show different average number of CySCs, which also depends on the temperature. Our initial analyses were based on data from Amoyel et al. 2014, where ~ 44 average CySCs were recorded. In the recovery from starvation experiment we found a different total number of CySCs. However, all the conclusions from the initial analyses are robust to changes in the total stem cell pool size.

Discussion of other paradigms:
Intestinal stem cell- the potential parallels are perhaps murky in light of recent evidence for (some of) the population previously considered TAs to be part of the stem cell pool. This should be referenced. It is arguable to what degree it is relevant to the current models.

© 2025. Published by The Company of Biologists under the terms of the Creative Commons Attribution License (https://creativecommons.org/licenses/by/4.0/).

Reply: The reviewer is correct that the observation of previously considered transit-amplifying (TA) cells de-differentiating during regeneration adds a more nuanced perspective to the division between TA cells and stem cells. However, we believe that, rather than complicating the analogy, these findings actually reinforce it: one could consider that cells previously considered as TA cells are somewhat similar to licensed CySCs, in that they have moved towards differentiation but remain capable of returning to the niche and resuming stem cell function. A de-differentiation event in a TA cell may be broadly analogous to a de-licensing event. We have expanded the relevant paragraph to include this discussion and provided references.

I think that another interesting paradigm deserves mention.
In essence, the key modeling features for heterogeneity in the testis among stem cells involve cell locations which differ by (a) division and (b) immediate conversion to a differentiated cell. The same type of organization (but opposite division rate polarity) exists for Drosophila ovarian FSCs. The situation there is well-documented experimentally but the question of how or if it has specific benefits has not, I believe, been addressed.

Reply: We thank the referee for this suggestion. The system in question was modeled in Kronen et al. (2014)(https://doi.org/10.1371/journal.pone.0101085). Their modeling focused on determining when neutral competition is the governing population dynamics and the conditions under which biased competition for niche access is triggered. As the reviewer correctly points out, the question of whether this tissue architecture confers robustness to perturbations, manages stochastic fluctuations, and prevents extinction remains to be addressed. We have added a reference to this system in paragraph 7 of the Discussion.

Summary:
I hope that the authors will make appropriate revisions in response to these comments. From my reviewer perspective, I trust those will be made and would not require that I find those responses satisfactory for acceptance. I would nevertheless be interested to see what the responses are, continuing what I believe has been a productive dialog for all.

Reply: We once again thank the reviewer for taking the time to engage with us in this dialogue.

Third decision letter

MS ID#: DEVELOP/2024/202796

MS TITLE: Licensing and Niche Competition in Spermatogenesis: Mathematical Models Suggest Complementary Regulation of Tissue Maintenance

AUTHORS: Rodrigo Garcia-Tejera, Jing-Yi Tian, Marc Amoyel, Ramon Grima, and Linus Schumacher

I am happy to tell you that your manuscript has been accepted for publication in Development, pending our standard publication integrity checks.

Reviewer 1

Advance summary and potential significance to field

The authors have amended the paper according to my requests satisfactorily. I am happy to support publication of this paper.

Comments for the author

No further suggestions.

© 2025. Published by The Company of Biologists under the terms of the Creative Commons Attribution License (https://creativecommons.org/licenses/by/4.0/).

Reviewer 2

Advance summary and potential significance to field

Previously discussed- no change (except that responding to the suggestions will likely increase value for experimentalist readers).

Comments for the author

I believe the value of papers like this is enhanced if they address and professionally acknowledge both mathematical and experimental research and researchers, even though the bulk of the content may be mathematical.
The last reviewing round offered some opportunities to broaden and deepen the critical analysis of relevant experimental data. For the most part, these resulted in little or no adjustment to the main text. In some cases, the reviewer points may have been mis-understood or did not connect with familiarity. I am therefore including in this review some suggestions for an outline of specific additions that could be made to improve the quality and value for experimental researchers (probably the major audience for Development).

1. A central issue is the return of somatic cells ("licensed cells") to hub contact.

At present, the Introduction says licensed cells "can also fully regain stem cell function should they get back in contact with the hub [16] [17]."
Minor criticism- these cells are already stem cells- what they regain is division.
More important- the key question is whether they do return.

Later, in the Results, the new sentence was added, "The frequent migration of licensed stem cells back to the niche is supported by previous work [40] and our lineage tracing experiment (refs to suppl. Material)."

The supplementary materials do contain an expanded description of the lineage experiment.

This, in my opinion, does not suffice. It says nothing about the method, any limitations or what "frequent" means in the main text. The experimental evidence for this central component of the model is important to a reader genuinely interested in stem cell biology. Those readers are not going to comb through supplementary materials, just in case there is sufficient further detail there- the key outline of methods and reservations need to be in the main text.

I suggest something along the lines of the following (the authors will have to determine where, in the main text, to place this).

CySCs not in contact with the hub selectively express spict-GAL4, allowing preferential activation of UAS-flp recombinase and consequent FRT recombination to initiate permanent GFP expression when a temperature-sensitive GAL80 is inactivated at the restrictive temperature. Cells labeled with GFP in this way were initially mostly not in contact with the hub but were previously found to accumulate next to the hub over time (ref). We repeated this experiment with a similar result (refs). In these experiments, some cells initially contacting the hub might be labeled, labeling is continuous and many cells per hub are labeled. Hence, it is not possible to estimate the frequency with which cells return to contact the hub, but this appears to be a common event. Indeed, this return to hub contact appears to be the underlying reason for CysSCs being maintained by population asymmetry despite CySC division generally producing exactly one daughter contacting the hub, and for all CySC daughters being considered part of the stem cell pool (refs).

Regarding this section in Supplementary materials, two comments:
(i) the genotype has hs-flp. This is not necessary and poses the potential danger of introducing unwanted recombination (flip-out is very sensitive). Are the authors sure hs-flp was present?

(ii) The citation of de la Maza et al., supporting DNA replication occurring in cells not touching the hub is interesting. However, I am wondering if it is correct. I could find no statement in that paper saying that for wild-type cells and the relevant figure does not point out such an example. Also, if

© 2025. Published by The Company of Biologists under the terms of the Creative Commons Attribution License (https://creativecommons.org/licenses/by/4.0/).

that were the case, there would be some obvious further questions. Are cyst cells trapped in G2? If not, is there a decision point in licensed CySCs whereby progress through S phase is linked to the decision to differentiate or return to the hub?

2. The value of this work will be greater if it is related to the general case or compared to other stem cell paradigms. It is of great value to discuss other paradigms with respect to what actually happens, according to experimental data (the discussion already has references to different types of modeling). The key issue for this paper is the existence of an intermediate, reversible, licensed state. Hence, my previous suggestion to comment on two paradigms in particular- Drosophila ovarian Follicle Stem Cells (FSCs) and mouse gut stem cells- the former because of a wealth of relevant information and the latter because it draws a lot of attention. The authors chose to say nothing about the former and little of relevance about the latter. More important, the reply of the authors and the citations included are woefully out of date on these subjects and therefore, in my opinion, miss the key points of my suggestions entirely. I therefore provide a suggestion of what I believe could usefully be said……….

Our study addresses the attributes of stability conferred on a stem cell community by systematic heterogeneity, wherein stem cells must proceed through a reversible licensed state before differentiating. There is significant experimental evidence for this arrangement for CysSCs and also for Follicle Stem Cells (FSCs) in the Drosophila ovary. FSCs, like CySCs, are governed by population asymmetry and are principally distributed among two anterior-posterior rings. Only the posterior FSCs can directly become Follicle Cells and are, in that sense, also licensed. The anterior and posterior FSCs also exchange positions, so that all behave as stem cells. As for CysSCs, FSCs differ in their cell cycling properties according to location. Here, it is the licensed posterior FSCs that divide several-fold faster than anterior FSCs. Anterior FSCs additionally can move anteriorly to become another differentiated cell type, Escort Cells, an activity that has some parallels to exchange between hub and CySCs in testes. The advantages of reversible heterogeneity and a licensed state may therefore also apply to FSCs. Interestingly, it is also clear that FSCs compete for niche space, which is defined by graded signals.

The mouse intestinal crypt has been a major focus of stem cell studies, where strongly Lgr5-positive cells had been considered the principal stem cells, maintained by population asymmetry. Moreover, it appeared that only the cells at the edge of the stem cell domain (at the “+3” position) could directly become Transit-Amplifying (TA) cells, so-named because of presumed inevitable later differentiation. It was also observed that Lgr5-positive cells could move both higher and lower in the crypt, suggesting again a reversible licensed state. Early studies favored net upward movement, suggesting that cells deeper in the crypt would, on average, survive longer. However, later studies contradicted those conclusions. Recent work also suggested that “TA” cells can move back into the Lgr5-high zone and constitute a major portion of the stem cell population. Thus, although the behavioral dynamics of these cells is not as clearly defined as for Drosophila CySCs and FSCs, there is also surely heterogeneity, a licensed state and reversible transitions to and from licensed states. Current evidence therefore suggests this is a general feature of the best studied population asymmetry paradigms.

3. This is a new comment, motivated by the theme of greater transparency for experimental methods & data.
Section C concludes that the variation in the two locations of CySCs seen in a specific (older) experiment is less than predict buy the first-generation model. It refers to a section in the methods, which quotes some average numbers. I think that the whole data set should be reproduced in this paper (as a supplement). It is not reasonable for readers to find it in an earlier publication (especially as there could be ambiguity about the chosen data and perhaps it is not cited in full there or in the simplest form). I suspect that the full data set would allow those specializing in statistics to make some of their own judgments about matches with model predictions.
In section D a better match is found by introducing niche competition. However, it is said that correspondence to experimental data requires a “carrying capacity”, N >50. This puzzles me. I believe that N corresponds to the maximum number of Zfh1-positive cells, which in various tests has been cited around 44 or 32 but never as high as 50. I think there should be some further explanation. Am I misunderstanding N or is the possibility of N being as high as 50 in reality an untested speculation (which should be stated)?

© 2025. Published by The Company of Biologists under the terms of the Creative Commons Attribution License (https://creativecommons.org/licenses/by/4.0/).

Along similar lines, I think it would be useful to say more about the economy and virtues of licensing (in the context of niche competition) by explicitly citing model predictions about (a) extinction and (b) variation for given numbers of stem cells (30, 50 etc.) for stem cells with or without a reversible licensing state. This sort of explicit statement of a few numbers is likely easier to grasp as a bottom-line for experimentalist readers than graphical or equation representations.

I hope that by submitting this review quickly it will be possible to improve the manuscript further (I am again interested in responses but not as arbiter) without delaying publication.

© 2025. Published by The Company of Biologists under the terms of the Creative Commons Attribution License (https://creativecommons.org/licenses/by/4.0/).
